# Cytokines and drugs targeting cytokines in Graves’ disease and thyroid eye disease

**DOI:** 10.3389/fimmu.2026.1800029

**Published:** 2026-08-13

**Authors:** Silvia Martina Ferrari, Giusy Elia, Francesca Ragusa, Eugenia Balestri, Chiara Botrini, Valeria Mazzi, Alessia Baglini, Licia Rugani, Giulia Del Carlo, Rocco Rizzi, Benedetta Cimbalo, Camilla Virili, Enke Baldini, Gilda Varricchi, Alessandro Antonelli, Poupak Fallahi

**Affiliations:** 1 Department of Surgical, Medical and Molecular Pathology and Critical Area, University of Pisa, Pisa, Italy; 2 Department of Medico-Surgical Sciences and Biotechnologies, Endocrinology Section, “Sapienza” University of Rome, Latina, Italy; 3 Department of Experimental Medicine, “Sapienza” University of Rome, Rome, Italy; 4 Department of Translational Medical Sciences (DiSMeT), University of Naples Federico II, Naples, Italy; 5 Center for Basic and Clinical Immunology Research (CISI), University of Naples Federico II, Naples, Italy; 6 World Allergy Organization Center of Excellence, University of Naples, Federico II, Naples, Italy; 7 Institute of Experimental Endocrinology and Oncology (IEOS), National Research Council (CNR), Naples, Italy; 8 Department of Translational Research and New Technologies in Medicine and Surgery, University of Pisa, Pisa, Italy

**Keywords:** autoimmune thyroid disorders, chemokines, cytokines, Graves’ disease, thyroid eye disease

## Abstract

Graves’ disease (GD) is an autoimmune disorder characterized by the production of stimulatory autoantibodies against the thyrotropin receptor, leading to hyperthyroidism and diffuse goiter. The most frequent extrathyroidal manifestation is Graves’ ophthalmopathy (GO), also known as thyroid eye disease (TED), which is responsible for significant morbidity due to orbital inflammation, tissue remodeling, and visual impairment. In recent years, increasing evidence has demonstrated that cytokines play a central role in both GD and TED, orchestrating immune cell activation, autoantibody production, fibroblast stimulation, and tissue remodeling. The evolving understanding of cytokine networks has reshaped disease models and paved the way for targeted therapies that interfere with specific immune pathways. We review the immunepathogenesis of GD and TED, the role of cytokine networks, and the available cytokine-targeted therapies.

## Introduction

Autoimmune thyroid disorders (AITD) include Graves’ disease (GD) and Hashimoto’s thyroiditis (HT), both of which are characterized by lymphocytic infiltration of the thyroid (1).

Clinically, patients with HT develop hypothyroidism, and the prevalence of HT is estimated to be between 5% and 10% worldwide (2).

Graves’ disease is the most common cause of hyperthyroidism in Western countries with near-normal iodine intake. The prevalence of GD is approximately 3% in women and 0.5% in men, and the peak incidence occurs between 30 and 60 years of age. The disease is more prevalent in women, with a female-to-male ratio of 5–8:1. Graves’ disease is characterized by the appearance of extrathyroidal manifestations, including Graves’ ophthalmopathy, or thyroid eye disease (TED), affecting approximately 30%–40% of patients with GD, GD dermopathy, affecting 2% of patients, and acropathy, affecting less than 1% of patients (1). Furthermore, patients with GD more frequently experience neuropsychiatric disturbances, reduced fertility, and pregnancy-related disorders.

The clinical presentation of GD is variable and can significantly impact patients’ overall wellbeing. Symptoms include tremor, heat intolerance, warm skin, weight loss despite normal eating habits, irritability, anxiety, goiter, menstrual irregularities, erectile dysfunction or reduced libido, palpitations, frequent bowel movements, fatigue, and other systemic features (3, 4).

In GD, pathogenic autoantibodies target the thyroid-stimulating hormone receptor (TSH-R) and are referred to as thyroid-stimulating hormone receptor antibodies (TRAb) (5).

Thyroid-stimulating hormone receptor antibodies can be classified as thyroid-stimulating antibodies (TSAb), thyroid-blocking antibodies (TBAb), or neutral antibodies (6, 7). TSAb are the pathogenic autoantibodies specifically associated with GD; they bind to the TSH-R on thyrocytes and induce cell proliferation, thyroid growth, and hypersecretion of thyroid hormones (T4 and T3) (6). TSAb can be detected by modern methods in approximately 99% of patients with GD.

Thyroid peroxidase antibodies (AbTPO) are present in 80% of patients and thyroglobulin antibodies (AbTg) in 60%, but neither is disease-specific (8).

Graves’ disease is a relapsing-remitting autoimmune disease characterized by acute phases of hyperthyroidism followed by periods of remission; however, hyperthyroidism can subsequently reappear after months or years after remission.

Thyroid eye disease can also occur in patients with euthyroidism and HT (approximately 2%) (9, 10). TED is an autoimmune disorder characterized by an orbital inflammation, with an incidence of 20–50 per 100,000 individuals per year (9, 11). TED is characterized by a wide spectrum of manifestations, including eyelid inflammation, proptosis, diplopia, and/or restrictive strabismus, and, in the most severe cases, exposure-related keratopathy, and dysthyroid optic neuropathy (5).

The majority of patients with TED (approximately 70%–75%) had a mild form of the disease, while moderate-to-severe TED occurs in 25% of patients and requires systemic therapy. A very severe form, characterized by optic neuropathy or severe exposure keratopathy, occurs in 2% of patients with TED.

Thyroid eye disease is characterized by the an initial acute phase followed by a progressive reduction in the severity of symptoms; however, recurrence of the acute inflammatory phase is also possible.

The target of the autoimmune process in TED is the orbital fibroblasts (OF), which expresses both the TSH receptor and the insulin-like growth factor 1 receptor (IGF-1R), which are co-localized on the surface of the membrane. TSAb and IGF-1R autoantibodies may activate the TSH/IGF-1 receptor complex on the OF, triggering intracellular pathways that induce fibroblast proliferation, differentiation into adipocytes, secretion of glycosaminoglycans (GAGs), and secretion of pro-inflammatory cytokines and chemokines that are associated with the appearance of the clinical manifestations (8).

In both GD and TED, the phenotype of infiltrating T lymphocytes may evolve over the course of the disease, with T helper 1 (Th1) cells predominating during the early stages and a shift toward a Th2-predominant immune response in patients with longer disease duration or during remission (12–14).

The cells of the target tissues (thyrocytes in GD and OF in TED) under the influence of Th1 cytokines, mainly interferon (IFN)-γ and tumor necrosis factor (TNF)-α, produce large amounts of Th1 chemokines, including chemokine (C-X-C motif) ligand (CXCL)-10, (CXCL)-9, and (CXCL)-11, together with other pro-inflammatory cytokines, including interleukin (IL)-6, CCL2, IL-8, and IL-21, which recruit other autoreactive immune cells in the target tissue, thereby initiating and perpetuating the autoimmune process (15).

Given the importance of cytokine and chemokines in the pathogenesis of GD and TED many studies have been conducted to evaluate their role in the immunepathogenesis of these diseases, with the additional goal of identifying novel cytokine- and chemokine-targeted therapies.

This narrative review summarizes the roles of cytokines and chemokines in GD and TED, together with new targeted therapies. The literature search was conducted using Pubmed with the following keywords: “Graves’ disease”, “hyperthyroidism”, “Graves’ ophthalmopathy”, “thyroid eye disease”, “cytokines”, “chemokines”, “INF-gamma”, “TNF-alfa”, “IL-2”, “CXCL10”, “CXCL9”, “CXCL11”, “CCL2”, “IL-6”, “IL-8”, “IL-21”, “Th1”, “Th2”, “Th17”, “targeted therapies in Graves’ disease”, and “targeted therapies in thyroid eye disease” from January 2005 to March 2026.

## Breakdown of immune tolerance in Graves’ disease

### Genetic and environmental determinants

In GD, the loss of immune tolerance is associated with the interplay of environmental triggers and genetic susceptibility. Environmental factors, such as stress, smoking, infections, iodine intake, and pregnancy, can affect immune responses and modulate disease expression. Polymorphisms in genes associated with immune regulation, including CTLA-4, human leukocyte antigen (HLA)-DR3, cytokine-related genes, and PTPN22, are important in abnormal immune activation (1). Cytokines and chemokines may guide the interplay between genetic predisposition and environmental risk factors. For instance, an excessive iodine intake can induce the appearance of an overt hyperthyroidism in predisposed patients with autoimmune thyroiditis (AT), while smoking increases the expression of pro-inflammatory cytokines and activates oxidative stress pathways in orbital tissues of patients with TED, thereby exacerbating the disease (1).

### Antigen presentation and T-cell activation

The presentation of TSH-R by antigen-presenting cells (APCs) to naïve T lymphocytes is associated with initiation of GD. The activation of autoreactive T lymphocytes induces macrophages, B cells, and dendritic cells to produce pro-inflammatory cytokines that reinforce the activation of other autoreactive T cells while contributing to the relative deficiency and dysfunction of regulatory T cells (Tregs), thereby allowing a sustained autoimmune process (16).

### T-helper cell subsets and cytokine profiles in Graves’ disease

In GD, the interaction between TRAb and cytokines creates a self-perpetuating autoimmune loop that drives thyroid overactivity and inflammation. In fact, the binding of TSAb to the TSH-R on thyrocytes triggers the activation of multiple signaling pathways, including nuclear factor κB (NF-κb), which is thought to activate the promoter regions of many chemokines (17).

T helper 1 cells are activated against thyroid antigens and TSH-R, producing large amounts of cytokines, such as IFN-γ and IL-2, which promote the activation of macrophage and upregulate the expression of MHC class II on the surface of thyrocytes, thereby increasing the presentation of thyroid antigens and perpetuating thyroid inflammation. IFN-γ and IL-2 are particularly important during the early acute phase of the disease because they induce the secretion of large amounts of Th1 cytokines (CXCL10, -9, and -11), which lead to the chemotaxis of additional Th1 cells, creating a self-amplifying inflammatory loop (18). Th1 cytokines act together with Th2 cytokines to activate autoreactive B-cells.

T helper 2 cytokines, such as IL-5, IL-4, and IL-13, promote B-lymphocyte proliferation and the production of autoantibodies. These Th2 cytokines induce the production of TSAb and other thyroid autoantibodies, and the Th2 response is predominant during the later phases of the disease and during remission (18).

The differentiation of Th17 lymphocytes is supported by cytokines, such as IL-1β, IL-6, and IL-23, and these cells secrete important cytokines, including IL-17A, IL-22, and IL-21 (19). Some studies have reported that IL-17 and inflammatory cytokines and chemokines play an important role in the pathogenesis of HT (20–23). Furthermore, IL-17 is important in patients with active TED, acting as a link between orbital inflammation and systemic autoimmunity (24).

Tregs play a central role in the maintenance of immune tolerance by secreting the anti-inflammatory cytokines IL-10 and transforming growth factor (TGF-β). In GD, many studies have shown a decrease in the number of Tregs, accompanied by functional defects. Reduced production of TGF-β and IL-10 contributes to an enhanced activity of T cells.

Moreover, patients with AITD show an increase in B regulatory cells, possibly to counteract the activation of Th17 inflammatory pathways (25).

The increased amounts of cytokines in the environmental thyroid tissue lead to the activation of thyrocytes themselves, which actively participate in the autoimmune response by secreting more chemokines and cytokines (26).

## Cytokine in Graves’ disease and thyroid eye disease

Interferon-γ enhances macrophage activation, antigen presentation, and chemokine production. In GD, IFN-γ sustains autoimmunity by upregulating the MHC class II expression on thyrocytes, transforming these cells into non-professional APCs. It also induces CXCL10 production, thus promoting the recruitment of further Th1 cells (27). In TED, IFN-γ activates OF and increases the expression of adhesion molecules. Its role is predominant in the early and active phase of TED, when it promotes CXCL10 production (28).

Macrophages, T cells, and fibroblasts produce the pro-inflammatory mediator TNF-α, which is critically involved in tissue damage, immune cell recruitment, and endothelial activation.

In GD, elevated serum TNF-α levels have been associated with disease activity, where it amplifies the autoimmune response and enhances MHC class II expression on thyrocytes (27). In TED, TNF-α activates OF, enhancing cytokine release, tissue remodeling, and GAGs production. Moreover, TNF-α amplifies inflammatory responses by acting synergistically with IL-1β and IFN-γ.

The important pathological role of TNF-α has led to the investigation of anti-TNF therapies in TED; however, results have been inconsistent, likely due to both disease heterogeneity and the redundancy of cytokine signaling pathways (28, 29).

## Interleukin-1 family

Interleukin-1β is a pro-inflammatory cytokine produced mainly by activated macrophages, monocytes, and fibroblasts following activation of the inflammasome (30, 31).

Interleukin-1β is present in the thyroidal tissue of patients with GD and promotes inflammation by inducing other cytokines, chemokines, and adhesion molecules. Moreover, IL-1β suppresses thyroid hormone synthesis and iodide uptake, linking immune activation to altered thyroid function (32).

In TED, IL-1β acts on OF by stimulating the synthesis of hyaluronan, resulting in tissue edema and orbital congestion. Furthermore, IL-1β stimulates the production of IL-6 and prostaglandins, amplifying inflammatory pathways. Therefore, IL-1β appears to play a central role in the pathogenesis of TED; however, to date, no clinical trials have explored this cytokine.

In addition, the less investigated IL-1α contributes to autocrine and paracrine inflammatory signaling, and similar to IL-1β, acts through the IL-1 receptor type I, activating NF-κb and MAPK pathways.

Interleukin-1 is modulated by IL-1 receptor antagonists (IL-1Ra), and the balance between IL-1α, IL-1β, and IL-1Ra constitutes an important modulatory aspect of a major inflammatory pathway that may play an important role in GD (33). The concentration of IL-1Ra in OF was significantly lower in patients with TED compared with controls. Li and Smith demonstrated that when stimulated by TSH, OF and fibrocytes from patients with GD produced IL-1Ra. Because of the lower concentration of IL-1Ra in the OF than in fibrocytes of patients with TED, OF reacted more significantly to IL-1β than fibrocytes did (34).

However, because of the redundancy and pleiotropy of IL-1 signaling, it is hard to obtain a targeted and specific therapeutic activity (34).

Interleukin-2 plays a key role in T-lymphocyte survival, proliferation, and in supporting the homeostasis of Tregs. Patients with AITD show alterations in IL-2 receptor expression and signaling that favor an expansion of effector T cells in GD (35).

## Interleukin-6 and the STAT3 Axis

Interleukin-6 is a pleiotropic cytokine produced by immune cells and by non-immune cells, such as endothelial cells, thyrocytes, and orbital fibroblasts (36). IL-6 stimulates the release of acute-phase proteins and has immune-regulatory properties by promoting T-cell and B-cell differentiation and maturation, in addition to activating cells such as fibroblasts (37, 38).

In GD, IL-6 leads to B-cell differentiation and antibody production, including TSAb, and high serum IL-6 levels correlate with disease severity and activity.

In TED, IL-6 acts through the JAK/STAT3 cascade promoting fibroblast survival, cytokine production, and resistance to apoptosis. Moreover, IL-6 sustains the immune dysregulation by promoting Th17-cell differentiation and suppressing Treg development.

Because of the central role of IL-6 in TED pathogenesis, targeting the IL-6 receptor is a strong rational therapeutic approach (37).

## Th17-related cytokines

In GD, increased circulating Th17 cells and elevated IL-17 levels have been reported, and these findings are particularly pronounced in patients with active disease and those with TED.

Interleukin-17 acts as a pro-inflammatory cytokine by stimulating the production of IL-6. In addition, IL-17 synergizes with other cytokines, including IFN-γ, IL-1β, and TNF-α, enhancing local inflammation (19, 39, 40). Moreover, IL-17 induces cytokine and chemokine production from fibroblasts and epithelial cells and upregulates IL-6 and IL-8 expression in the OF, thereby amplifying inflammatory feedback loops.

Both GD and TED are characterized by high expression of IL-23, which is essential for the stabilization and pathogenicity of Th17 cells. Therefore, the IL-23/IL-17 axis may represent a potential therapeutic strategy, particularly in refractory disease (41).

## Regulatory cytokines

Interleukin-10 is an anti-inflammatory cytokine produced by Tregs, macrophages, and B cells. It acts by suppressing pro-inflammatory cytokine production and limiting immune-mediated tissue damage. Reduced IL-10 levels or impaired IL-10 signaling have been associated with persistent immune activation and autoantibody production and are associated with disease activity in GD.

The regulatory cytokine TGF-β has a dual role in GD and TED; it supports the immune tolerance by promoting Treg differentiation, but also drives fibrotic processes in later stages of TED. Moreover, TGF-β induces fibroblast differentiation into myofibroblasts and promotes extracellular matrix accumulation in the orbital tissues, contributing to disease resolution during the early stages but also contributing to irreversible tissue remodeling in chronic TED (42, 43).

Therefore, cytokine expression in GD and TED varies during the course of the disease, and this aspect has important therapeutic implications, since anti-cytokine therapies are likely to be most effective during the early inflammatory, active phase of the disease (3) (**Table 1**).

**Table 1:** Disease-stage specific cytokine profiles.

Cytokines	Graves’ disease		Thyroid eye disease
IFN-γ	It is involved in early acute phase of GD. It induces the secretion of huge amounts of Th1 cytokines (CXCL10, -9 and -11), that attract additional Th1 cells creating a self-amplifying inflammatory loop (18). It upregulates the MHC class II expression on thyrocytes.		It activates OF and increases expression of adhesion molecules. It has a predominant role in the early and active phase of TED with the induction of CXCL10 production (28).
IL-2	It is involved in early acute phase of GD. It induces the secretion of huge amounts of Th1 cytokines (CXCL10, -9 and -11), that attract additional Th1 cells creating a self-amplifying inflammatory loop (18).		
Th1 dependent chemokines (CXCL10, -9 and -11)	It is involved in early acute phase of GD. CXCL10 was detected in thyrocytes, endothelial and inflammatory cells, which also expressed a large amount of CXCR3 (50–55). In vitro human primary cultures of GD thyrocytes are not able to secrete CXCL10; IFN-γ induces dose-dependently CXCL10; IFN-γ plus TNF-α induces a strong synergistic CXCR3 chemokines secretion (vs. IFN-γ alone) (48, 56). GD patients with recent disease onset show high CXCL10. Hyperthyroidism is associated with elevated Th1 chemokines, which are reduced by MMI. CXCL10 levels are similarly increased in newly diagnosed and relapsing patients, thus identifying the active phase of GD (57). Early GD is characterized by a predominant Th1 immune profile, which shifts toward Th2 during MMI therapy (58). CXCL10 levels decrease after radioiodine ablation or thyroidectomy, indicating the thyroid as the main source (59).		Elevated serum CXCL10 levels are found in the active phase of TED (1). In vitro studies on primary cultures from TED patients, retro-OF and preadipocytes showed that CXCL10 is secreted only after stimulation with IFN-γ in a dose dependent manner, and the effect was enhanced by TNF-α, suggesting a role for retrobulbar cells in sustaining orbital inflammation (62). Serum CXCL9 and CXCL10 levels decrease during corticosteroid and radiotherapy treatment in TED patients (63, 64).
RANTES/CCL5			IL-17A induces RANTES expression in OF from TED patients through CD40–CD40L interactions, further supporting the involvement of chemokines in TED pathogenesis (67).
CXCL8			Associated with the later chronic, TNF-α–dominant phase (56).
CXCL13			Aberrant CXCL13/CXCR5 signaling has been implicated in impaired B-cell homeostasis (67).
TNF-α	Elevated serum TNF-α levels have been associated with GD activity, where it amplifies the autoimmune response and enhances MHC class II expression on thyrocytes (27).		It activates OF, enhancing the cytokine release, tissue remodeling and GAGs production. Moreover, TNF-α amplifies the inflammatory responses by synergistically acting with IL-1β and IFN-γ (28, 29).
Th2 dependent chemokines (i.e IL-5, IL-4, and IL-13)	Th2 dependent chemokines activate autoreactive B-cell, and the production of autoantibodies, acting together with Th1 cytokines. Th2 response is predominant during the later phases of the disease and during remission (18).		
IL-1β	IL-1β is present in the thyroidal tissue of GD patients, and promotes inflammation by inducing other cytokines and chemokines and adhesion molecules; moreover IL-1β suppresses thyroid hormone synthesis and iodide uptake, linking immune activation to altered thyroid functions (32).		It acts on OF by stimulating the synthesis of hyaluronan with a consequent tissue edema and orbital congestion. Furthermore, IL-1β stimulates the IL-6 and prostaglandin production, amplifying the inflammatory pathways.
IL-1α and IL-1Ra	The balance between IL-1α, IL-1β, and IL-1Ra constitutes an important modulatory aspect of a major inflammation pathway that may play important roles in GD (33).		The concentration of IL-1Ra in OF is significantly lower in TED patients compared to controls.
IL-2	Patients with AITD showed alterations in IL-2 receptor expression and signaling that favor an expansion of effector T-cell in GD (35).		
IL-6	In GD, IL-6 leads to B-cell differentiation and antibodies production such as TSAb, and high serum IL-6 levels correlate with disease severity and activity (37).		In TED, IL-6 acts through the JAK/STAT3 cascade promoting fibroblast survival, cytokine production, and resistance to apoptosis. Moreover, IL-6 sustains the immune dysregulation by promoting the Th17 cell differentiation and suppressing Tregs development (37).
IL-17 and IL-23	In GD, increased circulating Th17 cells and elevated IL-17 levels have been reported, and these findings are particularly pronounced in patients with active disease and in those with TED. Both GD and TED showed high expression of IL-23, that is essential for the stabilization and pathogenicity of Th17 cells. Therefore, the IL-23/IL-17 axis may represent a potential therapeutic strategy, particularly in refractory disease (41).		
IL-10	Reduced IL-10 levels or impaired IL-10 signaling have been related to a persistent immune activation and autoantibodies production and is associated with disease activity in GD.		
TGF-β	It supports the immune tolerance by promoting Treg differentiation.	It supports the immune tolerance by promoting Treg differentiation, induces fibroblast differentiation into myofibroblasts and extracellular matrix accumulation in the orbital tissues, contributing to disease resolution in early stages but to irreversible tissue remodeling in chronic TED (42, 43).	

GD, Graves’ disease; MMI, methimazole; OF, orbital fibroblasts; TED, thyroid eye disease.

## Chemokines and immune cell recruitment

Chemokine refers to a particular subgroup of cytokines with chemotactic activity for different leukocyte subtypes (44).

They are peptides with low molecular weight and high sequence homology, classified into four main subfamilies (CXC, CC, CX3C, and XC), and they bind with G protein-linked transmembrane receptors localized on the surface of target cells (44). They attract leukocytes to inflamed sites, playing a role in inflammation, angiogenesis, tumor growth, and sclerosis (44–46).

The CXC chemokines inducible by IFN-γ – CXCL9, CXCL10, and CXCL11 – are associated with Th1-mediated immune responses (47), and they bind the CXC3 receptor (CXCR3) (48).

CXCL10 and its CXCR3 receptor play a pivotal role in the initial phases of AITD (49, 50).

In patients with GD, CXCL10 was detected in thyrocytes, endothelial cells, and inflammatory cells, which also expressed a large amount of CXCR3 (50–55).

*In vitro*, human primary cultures of GD thyrocytes do not secrete CXCL10; however, IFN-γ induces CXCL10 dose-dependently, and cotreatment with IFN-γ and TNF-α induces a strong synergistic secretion of CXCR3 chemokines (*vs.* IFN-γ alone) (48, 56).

Graves’ disease patients with recent disease onset showed high CXCL10 levels; the release of CXCL10 by follicular cells recruits Th1 lymphocytes into the gland (49), and the locally produced IFN-γ-dependent chemokines reach the circulation, achieving high circulating levels (1).

In GD, hyperthyroidism is associated with elevated Th1 chemokines, which are reduced by methimazole (MMI). CXCL10 levels are similarly increased in newly diagnosed and relapsing patients, thus identifying the active phase of GD (57). Early GD is characterized by a predominant Th1 immune profile, which shifts toward Th2 during MMI therapy (58). CXCL10 levels decrease after radioiodine ablation or thyroidectomy, indicating that the thyroid is the main source (59). CXCL10 polymorphisms are associated with disease severity, and persistently high levels may predict a lack of remission (60, 61).

As stated above, OF participate actively in the pathogenesis of TED (42). Elevated serum CXCL10 levels are also found during the active phase of TED (1). *In vitro* studies using primary cultures from TED patients, including retro-OF and preadipocytes, showed that CXCL10 is secreted only after stimulation with IFN-γ in a dose-dependent manner, and the effect is enhanced by TNF-α, suggesting a role for retrobulbar cells in sustaining orbital inflammation (62). Serum CXCL9 and CXCL10 levels decrease during corticosteroid and radiotherapy treatment in patients with TED, correlating with disease activity and supporting their potential use as biomarkers to guide therapy (63, 64).

Regulated upon activation, normal T-cell expressed and secreted (RANTES/CCL5) is a member of the CC chemokine family and a downstream target of NF-κB (65, 66). Wan et al. demonstrated that IL-17A induces RANTES expression in OF from patients with TED through CD40–CD40L interactions, further supporting the involvement of chemokines in TED pathogenesis. Aberrant CXCL13/CXCR5 signaling has also been implicated in impaired B-cell homeostasis, contributing to disease activity in TED (67).

Moreover, primary human cultures of fibroblasts and preadipocytes from patients with TED show differential secretion of CXCL8 and CXCL10, reflecting distinct disease phases: CXCL10 predominates in the early IFN-γ–driven phase, whereas CXCL8 is associated with the later chronic, TNF-α–dominant phase (56).

## Drugs targeting cytokines and cytokine signaling in GD and TED

### From cytokine biology to targeted therapy

**Figure 1:**
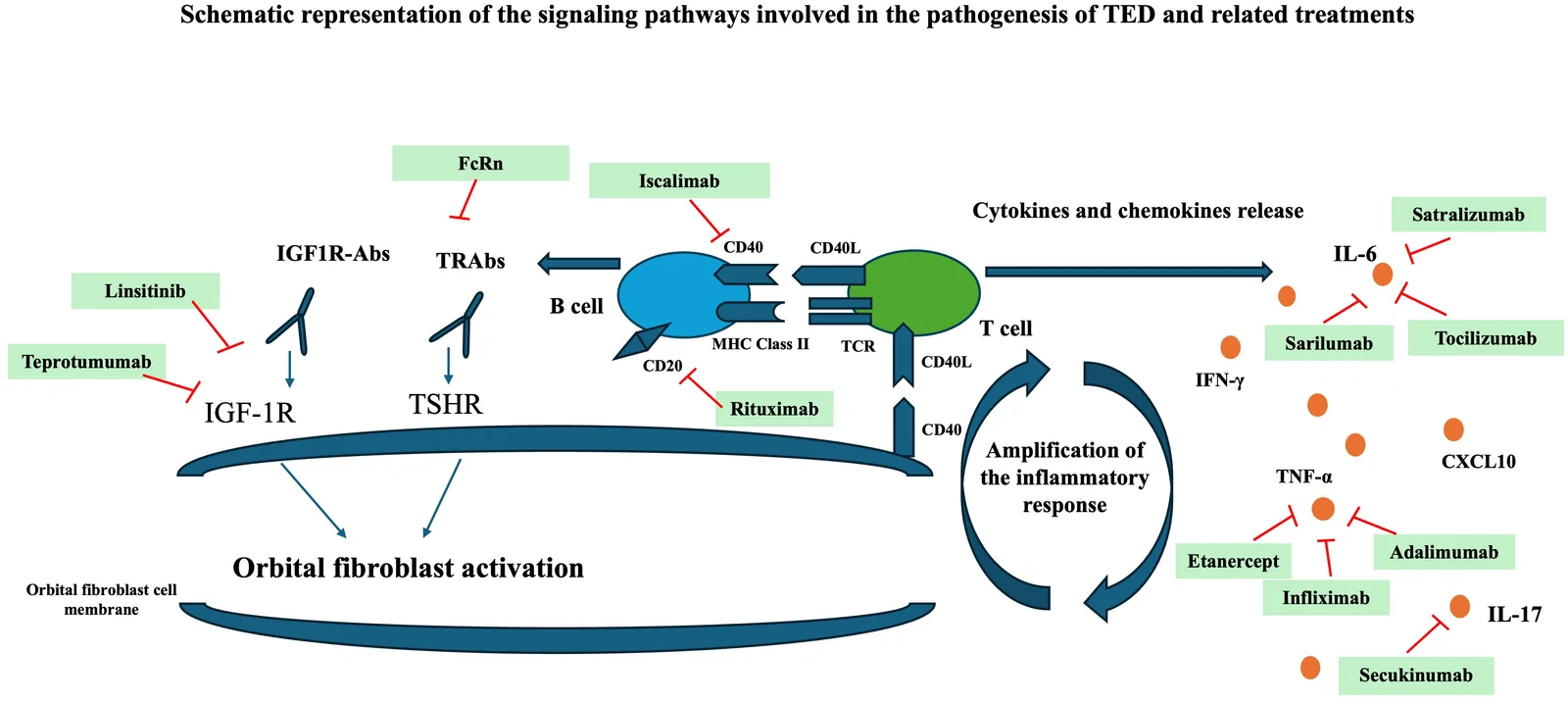
Schematic representation of the signaling pathways involved in the pathogenesis of TED and related treatments.

Knowledge of the important role of cytokines in immune dysregulation in GD and TED has transformed therapeutic paradigms. Traditional treatments, such as glucocorticoids (GCs) and antithyroid drugs (ATDs), are able to exert broad immunosuppressive effects but without selectively targeting the pathogenic pathways. Recent advancements in immunology have led to the development of new therapies able specifically target the involved cytokines and their downstream signaling pathways (**Figure 1**). New cytokine-targeted drugs have shown particular promise in TED compared with GD without ophthalmopathy due to the presence of thyrotropin receptor autoantibodies and the effectiveness of conventional treatments (**Tables 2**, **3**).

**Table 2:** Drugs targeting cytokines and cytokine signaling in GD.

Drug	Target	Clinical outcomes	Adverse events	Ref
Rituximab	CD20 antigens on B cells	A prospective study in a small number of patients suggested a beneficial effect in relapsing GD.		(84)
		A multicenter phase 2 clinical trial in patients with GD, evaluated RTX (along with antithyroid drug for 1 year), and after 2 years 48% of patients achieved the hyperthyroidism remission.	No serious side effects linked to treatment	(85)
		A prospective, controlled, nonrandomized study showed that RTX treatment may induce a sustained remission in patients with GD and low TRAb levels; whereas did not affect autoantibody levels and seems ineffective in patients with high TRAb levels.	Some patients had adverse events at the first infusion: hypotension, nausea, fever, chills, and tachycardia. Other AEs were: had serum sickness-like reaction with joint pain and fever after the second infusion; worsening of gastrointestinal symptoms. Two patients in each group (“RTX” and “no RTX”) had a minor infection during the first year of follow-up.	(86)
Iscalimab	CD40	An open-label trial in GD patients with untreated hyperthyroidism showed twenty-four weeks after the last dose that 47% of patients achieved euthyroidism with a relapse in 4/7 and a decrease of circulating TRAb levels.	All treatment-related AE were mild or moderate and resolved by end of the study; only 1 patient reported moderate cystitis.	(113)
ATX-GD-59	HLA-DR molecules on the surface of immature dendritic cells	A Phase I open label trial in 12 patients with previously untreated mild to moderate Graves' hyperthyroidism. Ten subjects received all doses of the drug; five of them had free triiodothyronine within the reference interval by the 18-week visit. Two further subjects had improved free thyroid hormones by the end of the study (7/10 responders), whereas 3 showed worsening thyrotoxicosis. Serum TSHR autoantibody concentrations reduced during the study and correlated with changes in free thyroid hormones.	The most common adverse events were: mild injection-site swelling and pain	(118)
Azathioprine	inhibits purine synthesis	A randomized, open-label, and parallel-group clinical trial investigated azathioprine as an adjuvant therapy to ATDs for moderate and severe GD showing that a combined therapy could be a promising approach to achieve rapid TSH control, lowering the relapse rate as well as significantly lowering the levels of TRAb compared with those who only received ATDs.	Incidence of side effects such as gastrointestinal disturbances, leucopenia, and infection was not statistically significant between the intervention and control groups.	(107)

ATD, antithyroid drugs; AE, adverse event; GD, Graves’ disease; RTX, rituximab; TRAb, thyrotrophin receptor antibodies.

**Table 3:** Drugs targeting cytokines and cytokine signaling in TED.

Drugs	Target	Clinical outcomes	Adverse events	Ref
Tocilizumab	IL-6	A systematic review report efficacy of TCZ in: -reducing TED inflammation during active TED (reduction of CAS of ≥3 points), and a relapse rate of 8.2%; -reducing proptosis	Common side effects: increased levels of liver enzymes, of plasma lipid. AEs were acceptable in most reports or studies. Breast and bladder cancer in one of the studies with 10 patients (the causal link between the 3 cases of cancer and TCZ treatment is uncertain). Another patient presented acute pyelonephritis.	(70)
		A recent meta-analysis has evaluated patients with steroid-resistant, active TED treated with intravenous TCZ showing CAS and proptosis reduction, a diplopia response in 48%, and reactivation rate only in 1%.	Most reactions were mild to moderate, and include gastrointestinal, pulmonary, and renal infections and headaches. Three patients had severe complications, such as: moderate increase in transaminase levels; acute pyelonephritis; anaphylactic shock with bronchospasm.	(71)
Sarilumab	IL-6	An observational case series abstract of 5 TED patients (active, moderate-severe TED) report that all patients achieved a mean CAS reduction of 3 point, and an improvement of disease severity and antithyroid antibody levels.	No AEs were recorded except for mild neutropenia in one patient, not requiring dose reduction. No patient required subsequent immunosuppressive treatment or surgery.	(73)
		A retrospective study showed: a significant reduction of CAS and of thyroid stimulating immunoglobulin. The GO-QOL baseline mean total score significantly improved. Sarilumab appeared as an effective and safe option for the treatment of low CAS TED.	The most frequent AEs were: neutropenia, hypercholesterolemia, thrombocytopenia, hypertransaminasemia, asthenia.	(74)
Satralizumab	IL-6	Two double-masked, placebo-controlled, multicenter phase 3 trials evaluated the drug in TED patients with active, moderate-to-severe, or chronic inactive TED hypothesizing important reduction of CAS [(SatraGO-1 (NCT05987423); SatraGO-2 (NCT06106828); respectively].	The main risks observed are rash, arthralgia, a decrease in neutrophil count and elevated liver enzymes	(77)
Infliximab	TNF-α	Beneficial effect of IFX in a patient with active TED refractory to steroid, with a significant reduction in inflammatory activity index.	No short-term side effects reported	(81)
		Case report of a patient with active TED and optic nerve neuropathy, with therapeutic success in the form of reduction of CAS and disappearance of optic disc oedema 72 hours after the drug application.	No AEs reported.	(80)
Etanercept	TNF-α	A pilot study presented 10 patients with mild to moderate TED showing a significantly reduction of the inflammatory activity.	3 patients had a recurrence of TED after discontinuing ETA	(79)
		Case report of a patient with TED and rheumatoid arthritis in treatment with ETA in combination with methotrexate. The patient reported a reduction of: exophthalmos; CAS; the oculomotor muscle oedema; TRAb levels.	No adverse effects reported.	(78)
Adalimumab	TNF-α	A retrospective study in 10 patients with active phase of TED showed: 6/10 a reduced inflammation, while 3 an increase, and one remained unchanged. No significant change in the CAS index after 3 months of treatment. Analysing separately the 5 patients with high baseline CAS index (> 4) showed a significant regression, and four of the 5 patients also reported subjective improvement in TED symptoms. No improvement in proptosis or ocular motility disturbances.	One patient experienced a significant complication (hospitalisation for sepsis).	(82)
Rituximab	CD20 antigens on B cells	A double-blind, randomized trial comparing RTX with ivMP in patients with active moderate to severe TED. RTX showed superiority over iv glucocorticoids in reducing disease activity.	Major side effects reported after ivMP were: increase of aminotransferases, increase of γ-glutamyl transferase and glycemia and glycosylated hemoglobin. Two patients treated with RTX had a major infusion reaction, likely a cytokine release syndrome, with a rapid onset of orbital edema and ensuing decrease of vision. Minor side effects observed in ivMP patients were: hypotension, dyspepsia, insomnia, and mild mood disorders. RTX patients showed mild infusion reactions, such as throat itching and nose stuffing. One RTX patient had transient hypotension.	(89)
		RTX failed to show benefit over placebo in patients with active and moderate to severe TED.	AEs reported were: myalgias/arthralgias; rash, itching; infectious (bronchitis, conjunctivitis); vasculitis; optic neuropathy; severe lacrimation; gastrointestinal disorders	(90)
		A low‐dose of RTX may be effective in the amelioration of inflammatory activity in patients with an active TED resistant to steroid; thus, reducing systemic steroid administration	Low-dose RTX may achieve long-term inactivation of TED and an improved safety profile.	(91)
		A retrospective audit showed that a low-dose of RTX is an efficacious, well-tolerated and safe treatment for active TED; reducing disease activity and allowing reduced administration of systemic steroid.	A transient infusion-related rash.	(92)
Tofacitinib	JAK1/3 inhibitor	Improvement of the inflammatory signs and symptoms in patients with refractory TED.		(96)
Mycophenolate Mofetil	inhibits de novo purine synthesis	The combination of MMF and glucocorticoids in the treatment of moderate to severe TED showed better clinical results (such as improvement of CAS and QOL score, visual acuity, intraocular pressure, and others parameters) than iv shock with glucocorticoids alone.	The main reported AE were: mild abnormal liver function and menstrual disorders in the control group, with no differences between control or experimental group. The combination of MMF did not increase the number of significant adverse effects.	(99)
		A retrospective cohort study of TED patients with active moderate/severe to sight-threatening TED showed that MMF as an effective and safe second-line immunosuppressive agent.	Significant side effects occurred in 2 patients (viral pneumonia and GI upset), that had also other comorbidities, then these events may not necessarily be related to MMF.	(100)
		A retrospective study in moderate-to-severe TED patients showed that if the TSI levels are lower it is better a first-line IVMP monotherapy, sparing potential side effects of MMF; in presence of elevated TSI patients may benefit from dual therapy of ivMP and mycophenolate.		(102)
		A prospective, non-randomized, interventional case series suggested that mycophenolate mofetil plus low-dose prednisolone can be introduced as a new optimal dosing regimen in TED because of its better effect on chronic complications such as proptosis and diplopia.	AEs occurred in six of 261 patients: impaired liver function, and gastrointestinal complications.	(103)
Azathioprine	inhibits purine synthesis	It resulted no effective for the treatment of patients with moderately severe TED.		(105)
		A multicentre, double-blind, randomised controlled trial suggested that, if tolerated, azathioprine might improve 48 week clinical outcomes in patients with active moderate-to-severe TED. This finding supports the use of long- term antiproliferative treatments as steroid-sparing therapies in combination with systemic corticosteroids for the treatment of active moderate-to-severe TED.	The most common AEs were mild infections.	(106)
Cyclosporine	calcineurin	A single-blind, randomized clinical trial compared prednisone with cyclosporine showing that single-drug therapy with prednisone is more effective than cyclosporine in patients with severe TED. A combined treatment can be effective in patients not responding to either drug alone.	Side effects in the cyclosporine group were an increase of the mean blood pressure; rise of plasma creatinine concentration in one patient. The number of minor side effects were similar between the two groups, while moderate and major adverse effect occurred mainly during prednisone treatment.	(108)
Teprotumumab	IGF-1R	A multicenter, double-masked, randomized, placebo-controlled trial (NCT01868997) showed clinical benefit of teprotumumab in patients with active, moderate-to-severe TED, that registered an improvement in proptosis, CAS and QOL.	The majority of AEs were mild and resolved while the patients continued to receive the intervention. Hyperglycemia, the only AE clearly identified as related to teprotumumab.	(123)
		A randomized, double-masked, placebo-controlled, phase 3 multicenter trial OPTIC Study (NCT03298867) investigated teprotumumab in patients with active, moderate-to-severe TED. Teprotumumab resulted in better outcomes with respect to proptosis, CAS, diplopia, and QOL than placebo	Most AEs were mild or moderate in severity; two serious events occurred in the teprotumumab group, of which one (an infusion reaction) led to treatment discontinuation. AEs of special interest were: potential infusion-related reactions, muscle spasms, hyperglycemia, diarrhea, and hearing impairment.	(124)
		An Open-Label Clinical Extension Study conducted in TED patients who previously received placebo or who flared in the OPTIC study. The study showed that patients with long-standing TED responded similarly to those treated in the early stages of the disease; those patients with an insufficient initial response or flare may benefit from additional teprotumumab therapy.	AEs in the group of patients treated for the first time. All AEs were mild or moderate, such as: potential infusion reaction; hyperglycemia; new-onset type 2 diabetes mellitus; hearing impairment. No patients experienced a serious AE. AEs in the group of re-treated patients. All were mild and moderate, such as: potential infusion reactions; hearing impairment. No AEs associated with hyperglycemia were reported during treatment.	(125)
		Randomized double-masked, placebo-controlled trial in chronic/low disease activity TED, showed significantly improved proptosis with respect to placebo.	The most frequently mild to moderate reported AEs in the teprotumumab group were: muscle spasms; fatigue, headache, dry skin, eye pain and pruritus, Hb1Ac increased and hypertension.	(126)
Linsitinib	IGF-1R	A phase 2b/3, randomized, double-masked, placebo-controlled study in active, moderate to severe TED showed proptosis reduction as its primary endpoint.	The majority of AEs were either mild or moderate, such as: hyperglycemia, diarrhea, headache, nausea, fatigue, ALT and AST increase, hyperhidrosis, and muscle spasms.	(131)
Batoclimab	FcRn	A randomized clinical trial with TED patients showed a decrease of total circulating IgG and TRAb, however CAS and proptosis were not significantly improved after 12 weeks.	Most AEs were mild or moderate. Increases in LDL-C levels were likely related to reductions in serum albumin, both reversible following treatment discontinuation	(133)
Secukinumab	IL-17A	A randomized, double-blind, placebo-controlled, parallel-group, multicenter trial in patients with active, moderate-to-severe, non-sight-threatening TED showed: 1) No clinically meaningful changes were observed in ophthalmic symptoms and signs, proptosis, lid aperture, eye muscle motility, CAS, and health-related QOL; 2) No meaningful impact on serum levels of thyroid-related hormones and antibodies was observed	Well tolerated, with mostly mild AEs.	(24)

ADA, adalimumab; AE, adverse event; ALT, alanine aminotransferase; AST, aspartate aminotransferase; BAFF, B-lympocyte-activating factor; CAS, clinical activity score; ETA, etanercept; GI, gastrointestinal upset; GO-QOL, Graves' Ophthalmopathy Quality of Life; IFX, infliximab; IL, interleukin; ivMP, intravenous methylprednisolone; MMF, mycophenolate mofetil; RCT, randomized clinical trial; RTX, rituximab**;** TCZ, tocilizumab; TED, thyroid eye disease; TNF, tumor necrosis factor; TRAb, Thyrotrophin receptor antibodies; TSI, thyroid stimulating inhibitor.

### Glucocorticosteroids

Glucocorticosteroids have pleiotropic immunosuppressive effects through the inhibition of NF-κB and activator protein-1 (68), leading to the reduction of several pro-inflammatory cytokines, including IFN-γ, TNF-α, IL-1β, and IL-6.

Moreover, they impair leukocyte trafficking, induce apoptosis of activated lymphocytes, and decrease vascular permeability. In the OF, glucocorticosteroids suppress the synthesis of GAGs and the production of inflammatory mediators.

Therefore, GCs are widely used for the treatment of active, moderate-to-severe TED, with the intravenous route of administration preferred over oral administration because of its greater efficacy and more favorable safety profile. However, some limitations are present, including the fact that 30%–40% of patients do not respond to the therapy or relapse, and the use of GCs leads to cumulative toxicity that limits their prolonged and/or repeated use (69).

Because of these limitations, the need for new specific drugs acting against pathogenic cytokine pathways is compelling.

### Anti–IL-6 therapy

As discussed above, IL-6 plays a crucial role in the cytokine network of TED by promoting B-cell activation, Th17 differentiation, and fibroblast survival while simultaneously inhibiting regulatory immune mechanisms (37).

### Tocilizumab

The humanized monoclonal antibody tocilizumab targets the IL-6 receptor, leading to the inhibition of both classical and trans-signaling pathways, and is used to treat inflammatory diseases such as rheumatoid arthritis.

A recent review showed that tocilizumab is most effective in reducing TED inflammation during active TED, with a reduction in CAS of ≥3 points and a relapse rate of 8.2% (70). Moreover, several studies showed its efficacy in reducing proptosis (70). A recent meta-analysis has evaluated patients with steroid-resistant, active TED treated with intravenous tocilizumab, showing CAS and proptosis reduction, a diplopia response rate of 48%, and a reactivation rate of only in 1% (71). However, one of the limitations of studies of tocilizumab is the lack of a treatment-naïve population; indeed, most studies have evaluated the drug in patients who failed previous therapy with GCs (37).

Moreover, a careful monitoring of TED patients receiving treatment with tocilizumab needs to be done, as it can increase the risk of infections and laboratory abnormalities, such as elevated liver enzymes and neutropenia.

### Sarilumab

Another anti-IL-6 receptor Ab is Sarilumab; it was approved for the treatment of rheumatoid arthritis and polymyalgia rheumatica (72).

Sarilumab has been studied (200 mg subcutaneously every 15 days for 11 months) in an observational case-series abstract involving five TED patients (active, moderate-severe TED). All patients achieved a mean CAS reduction of 3 points, along with improvements in disease severity and antithyroid antibody levels (37, 73).

Moreover, a retrospective study showed that sarilumab is an effective and safe option for the treatment of low CAS TED, reporting a significant reduction in CAS, thyroid-stimulating immunoglobulin levels, and an improvement in the baseline mean GO-quality-of-life (QOL) total score (74).

### Satralizumab

Satralizumab, a recombinant, humanized anti-IL-6 receptor monoclonal antibody, was approved for the treatment of neuromyelitis optica spectrum disorder (NMOSD).

Recently, satralizumab has been evaluated in patients with TED with active, moderate-to-severe, or chronic inactive disease in two double-masked, placebo-controlled, multicenter phase 3 trials [SatraGO-1 (NCT05987423) and SatraGO-2 (NCT06106828); respectively], with the hypothesis that it would reduce CAS.

The main risks observed are rash, arthralgia, a decrease in neutrophil counts, and elevated liver enzymes (75–77).

## Tumor necrosis factor inhibitors

The important role of TNF-α in inflammatory signaling has made its inhibitors, such as etanercept and infliximab, among the first biologics explored in TED. These monoclonal antibodies against TNF-α are able to reduce downstream cytokine production and leukocyte recruitment.

A case report of a patient with TED and rheumatoid arthritis treated with etanercept in combination with methotrexate showed an improvement in TED symptoms (78).

Different studies have demonstrated the efficacy of infliximab and etanercept in reducing inflammation in active TED patients resistant to steroid treatment (79–81).

Another anti‐TNF‐α agent is adalimumab, which has demonstrated anti‐inflammatory and steroid‐sparing effects in patients with TED (11, 82).

However, the efficacy of a single-cytokine TNF blockade is limited, likely because of the redundancy of cytokine networks and the presence of multiple inflammatory pathways, and TNF inhibitors are not routinely recommended for GO.

## B-cell depletion and indirect cytokine modulation

### Rituximab

Rituximab is a monoclonal antibody (chimeric murine/human) targeting the CD20 antigen on B cells, thereby inducing B-cell depletion. It was approved by the FDA for several diseases such as granulomatosis with polyangiitis, rheumatoid arthritis, chronic lymphocytic leukemia, and non‐Hodgkin lymphoma, and has also been used as an off‐label drug in different autoimmune diseases (43). Rituximab is not a cytokine inhibitor but indirectly modulates cytokine networks by reducing cytokine release.

Hyperthyroid patients treated with rituximab in combination with ATDs showed a significant reduction in TSAbs (83). The efficacy of rituximab in GD is still under research, and a prospective study in a small number of patients suggested a beneficial effect in relapsing GD (84).

A multicenter phase 2 clinical trial of 27 patients with GD evaluated rituximab (a single dose of 500 mg combined with ATDs for 1 year), and after 2 years, 48% of patients achieved remission of hyperthyroidism (85).

In a further study, rituximab was administered to 20 patients with GD receiving short-term MMI therapy, and the outcomes were compared with those of patients treated only with methimazole. In the rituximab group, after 24 months, remission was observed in 40% of patients, particularly in patients with lower TRAb levels at presentation (lower than 5 UI/L) (86). However, these results were not confirmed by other studies (84, 86–88).

Rituximab has also been proposed for the treatment of TED. However, randomized controlled trials evaluating rituximab in TED have shown conflicting results. It showed superiority over intravenous GCs in reducing disease activity, while in another study failed to show a benefit over placebo (89, 90). These discrepancies may be due to differences in patient selection criteria, disease duration, and baseline activity. An early use of low‐dose rituximab may be effective in the amelioration of inflammatory activity in patients with active TED resistant to steroids, thereby reducing the need for systemic steroid administration (91, 92).

The most frequent adverse effects of rituximab registered in RCTs for GD and TED were nausea, nasal stuffiness, throat itching, and slight temperature elevation (84–88, 93), with rare cases of ulcerative colitis, articular adverse events (94), and progressive multifocal leukoencephalopathy (79, 95).

## Janus kinases inhibitors

Janus kinase (JAK) inhibitors are a novel therapeutic class that blocks JAK/STAT signaling, thereby interfering with several inflammatory pathways downstream of multiple cytokine receptors, such as those for IFN-γ, IL-6, and the common γ-chain cytokines.

Tofacitinib, a JAK1/3 inhibitor, was shown to improve inflammatory signs and symptoms in patients with refractory TED (96). However, current evidence regarding JAK inhibitors in patients with TED is limited to case reports and small series, thus controlled clinical trials are needed.

## Other immunomodulatory agents affecting cytokines

### Mycophenolate mofetil

Mycophenolate mofetil (MMF) is an ester of mycophenolic acid (97) that inhibits *de novo* purine synthesis, leading to antiproliferative effects on T and B lymphocytes, suppression of the cell-mediated immune response, and antibody formation. Furthermore, MMF inhibits the recruitment of lymphocytes and monocytes in the inflamed site and the expression of adhesion molecules (98). MMF is used as a second-line immunosuppressive agent, particularly for the treatment of active TED and in moderate to sight-threatening cases. Patients treated with MMF showed a reduction in inflammation, with decreased TRAb and IL-6 levels, improved QOL scores, and decreased CAS scores (99). Some patients showed a long-sustained improvement even after 72 weeks of treatment (100). Mycophenolate mofetil is a relatively safe drug, and its most common side effects, such as headaches, difficulty sleeping, gastrointestinal symptoms, dizziness, rashes, and tremors, are not severe and are easily manageable. However, the most serious side effects are related to its immunosuppressive properties, which weaken the immune system (101).

Some studies investigated the efficacy of the combination of MMF with systemic GC treatment with respect to conventional treatment using only GCs. Patients also receiving MMF showed more effective control of local inflammation. Moreover, a higher incidence of adverse events was observed in those treated only with corticosteroids than in other treatment groups (99, 100, 102, 103). The MMF treatment of TED patients showed improved outcome compared with prednisone, with fewer adverse effects (103). The combination of MMF and GCs in TED treatment showed higher clinical efficacy than GC treatment alone (101, 104).

Azathioprine, a cytostatic agent that inhibits purine synthesis, was investigated in an earlier prospective study in patients with TED, which showed no beneficial effects in severe TED (105). In a recent RCT, azathioprine was administered together with oral prednisolone, demonstrating improved outcomes compared with patients treated with oral prednisolone alone. However, a large number of patients withdrew from the study because of drug-related adverse events (106).

A randomized, open-label, parallel-group clinical trial investigated azathioprine as an adjuvant therapy to ATDs for moderate and severe GD, showing that a combined therapy could be a promising approach to achieve rapid TSH control and an effective early reduction in TRAb levels (107).

Cyclosporine inhibits calcineurin, preventing the secretion of IL-2 by CD4+ T lymphocytes and thereby interfering with the expansion of lymphocyte clones. It is used to treat psoriasis, rheumatoid arthritis, Crohn’s disease, and to prevent organ transplant rejection. It is also used as eye drops in keratoconjunctivitis sicca (1 mg/mL eye drops, emulsion) in the treatment of severe keratitis in adult patients with dry eye syndrome (DES) who have not improved despite the use of artificial tears.

A study showed that cyclosporine was only minimally effective as a single agent in TED patients but suggested that it may have a role in combination with GCs for patients not responding to GCs alone (108, 109). Adverse effects associated with cyclosporine include increased plasma creatinine levels and, more commonly, hypertension.

Therefore, the combination of non-specific immunosuppressants, such as MMF, azathioprine, and cyclosporine, with steroid therapy appears useful for achieving stable results in the long term. However, their efficacy is limited, and their use is restricted because of their related toxicity profiles, including bone marrow suppression, nephrotoxicity, and hepatotoxicity (109).

### Radiotherapy

Orbital radiotherapy is able to reduce inflammatory cell infiltration and cytokine production within orbital tissues through a mechanism that is not yet completely understood. By modulating local cytokine networks, orbital radiotherapy may enhance responsiveness to immunosuppressive therapies (110).

## Cytokine-targeted therapies in Graves’ disease without ophthalmopathy

The current treatment of GD is based on the use of ATDs as the first-line therapy for GD in Europe, whereas radioiodine is used more commonly in the United States (111). MMI, carbimazole, and propylthiouracil (PTU) block the synthesis of thyroid hormones by inhibiting TPO. Propylthiouracil also blocks extrathyroidal deiodination of T4 to T3. After MMI and PTU therapy is discontinued, there is an elevated risk of recurrence (9). It is recommended that patients treated with ATDs be monitored during treatment for white cell counts and liver function tests (8, 112).

Over the past decades, patients with GD have been extensively treated with radioiodine because it provides relief from the symptoms of hyperthyroidism within weeks; however, it can cause or worsen ophthalmopathy. Thyroidectomy is generally the treatment of choice for patients with GD who have a large goiter, nodular thyroid disease, the presence of suspicious nodules, inadequate response to ATDs, a preference to avoid radioiodine treatment, and severe side effects of ATDs. It may also be performed during pregnancy if necessary according to the above-mentioned points (8, 9). New treatments are under investigation, including drugs targeting CD40-CD40L, FcRn, BAFF, and HLA-DR3.

Iscalimab is an anti-CD40 monoclonal antibody inhibiting the CD40–CD40L co-stimulatory signaling. CD40 (expressed on APCs) binds to CD40L on the T-cell membrane, leading to a co-activation pathway producing B-cell proliferation, germinal center formation, and immunoglobulin class switching (16). Iscalimab was investigated in an open-label trial involving 15 patients with GD and untreated hyperthyroidism who received the drug over a 12-week period (113). After 24 weeks of the last dose, 47% of patients achieved euthyroidism, with relapse occurring in 4 of the 7 responders, and a decrease in circulating TRAb levels in all patients. However, considering the small sample size, new clinical trials are needed (113).

Belimumab, a monoclonal antibody that targets the B-lympocyte activating factor (BAFF) (a humanized IgG1), is able to decrease B-cell survival and proliferation (114). Patients with GD have showed increased circulating BAFF levels, which were positively correlated with circulating TRAbs (115). An *in vivo* study using a GD mouse model showed that blocking BAFF mitigated hyperthyroidism (116), suggesting the need for further clinical studies in GD patients.

ATX-GD-59 is a peptide-based immunotherapy that includes two soluble synthetic peptides derived from the human TSHR sequence. ATX-GD-59 binds to HLA-DR molecules on the surface of immature dendritic cells, reducing the activation of APCs (117).

An open-label phase I study with 10 GD patients investigated ATX-GD-59; half of the treated patients achieved free triiodothyronine levels within the reference range, and serum TRAb concentrations decreased over the course of the study (16, 118).

## Therapeutic challenges and unmet needs

Cytokine-targeted therapies in GD and TED have to face different challenges, including the difficulty in defining optimal treatment windows, disease heterogeneity, the lack of predictive biomarkers, and concerns regarding long-term safety. Moreover, targeting a single cytokine may be limited due to the redundancy and plasticity of cytokine networks. More durable disease control may be obtained by combining different therapies or through upstream intervention.

### Teprotumumab

As stated above, IGF-1R is overexpressed on OF and immune cells in patients with TED. IGF-1R activation amplifies the inflammatory pathway in the orbital tissue by promoting the expression of pro-inflammatory cytokines, such as IL-8 and IL-6, and by enhancing fibroblast proliferation and adipogenic differentiation. IGF-1R is therefore a master regulator of cytokine-driven pathology in TED, and its signaling leads to reduced activation of intracellular pathways such as PI3K/Akt and MAPK, which are involved in cytokine transcription and fibroblast survival. Thus, inhibiting IGF-1R leads to suppression of multiple cytokines involved in TED pathogenesis (8).

Teprotumumab is a fully human monoclonal antibody that binds IGF-1R, disrupting the IGF-1R/TSHR signaling complexes and thereby reducing downstream inflammatory and adipogenic responses. Teprotumumab was first approved by the FDA in the USA for the treatment of TED (119). It was later, in 2024, approved in Japan and, in 2025, approved in the United Kingdom and Europe (120–122).

Two randomized, double-blind, placebo-controlled trials, the Phase 2 Trial TED01RV NCT01868997 (123) and the Phase 3 Trial HZNP-TEP-301 (OPTIC Study) NCT03298867 (124), investigated teprotumumab in patients with active, moderate-to-severe TED. These studies showed the efficacy of teprotumumab in improving CAS, proptosis, diplopia, and QOL of patients.

The reduction of proptosis observed with teprotumumab is greater than that typically achieved with GCs or other immunomodulatory therapies, and the OPTIC-X extension showed a sustained improvement in patients with relapsing or non-responsive TED (89.2%). At follow-up (Week 48), a high percentage of responders maintained their proptosis response (–3.5 ± 1.7 mm), CAS of 0 or 1, and diplopia response (125). Moreover, another RCT confirmed its efficacy in chronic TED, with mean proptosis improvement being greater in the teprotumumab group (–2.41) than in the placebo group (–0.92), leading to an extension of the FDA indication to include TED of any activity or duration (126).

Imaging and real-world data showed orbital volume reductions (127) and dysthyroid optic neuropathy improvement in up to 88% of cases (128). However, up to two-thirds of patients relapse within one year; therefore, retreatment or combined treatment with surgery could be needed (125, 127).

Teprotumumab showed an acceptable safety profile; however, different adverse events may occur, including hyperglycemia, hearing-related symptoms, muscle spasms, and gastrointestinal symptoms. Overall, the benefit–risk balance of teprotumumab is favorable and has improved TED management; however, its optimal application requires careful patient selection (29).

Currently, new molecules targeting IGF-1R, namely, veligrotug and linsitinib, are under investigation (129–131).

### Thyroid-stimulating hormone receptor antagonists

A new promising targeted therapy for GD and TED is the monoclonal antibody K1–70™ against TSH-R (132). In a first open-label phase I study involving 18 patients with GD, K1-70™ was associated with improvements in disease symptoms, including better mental concentration and sleep, reduced tremor and urinary urgency, and amelioration of TED manifestations, such as photosensitivity and exophthalmos (133). A phase 1 dose-escalation study evaluated K1-70™ in 12 patients with GD across four dose cohorts. The treatment showed a favorable safety profile and a significant reduction in TSAb levels across all cohorts, indicating effective blockade of TSH receptor signaling. Improvement in proptosis was reported in 50% of the eyes, indicating the potential of K1-70™ as a therapeutic option for inactive TED (134).

Recently, a novel compound, SYD5115, was evaluated *in vitro* and demonstrated nanomolar potency in blocking TSH-R in cells overexpressing TSH-R (135).

Another study investigated a different approach by evaluating a cyclotriazadisulfonamide compound (VGD040) that, in cell culture of human thyrocytes, was able to inhibit the translocation of TSHR from the endoplasmic reticulum to the cell membrane (136). In animal models, this approach showed a decrease in T4 values in response to TSH stimulation (137).

## Emerging therapies targeting cytokine pathways

### FcRn inhibitors

FcRn protects IgG from lysosomal degradation, prolonging its half-life. Therefore, FcRn inhibitors reduce circulating IgG levels, decreasing autoantibody-mediated immune activation and representing a promising strategy for autoimmune diseases, including GD and TED (138).

Batoclimab is a human anti-neonatal Fc receptor (FcRn) mAb that, by blocking FcRn-IgG interactions, leads to the degradation of autoantibodies, including TRAbs. In a recent randomized clinical trial, the effectiveness of batoclimab was studied in TED patients (139) who were treated with a subcutaneous injection of the drug. Patients showed decreases in total circulating IgG and TRAb; however CAS and proptosis were not significantly improved after 12 weeks (139). These results are still preliminary; however, the decrease in TRAbs suggests its possible use in the management of GD. Multicenter clinical trials of batoclimab in patients with GD are currently ongoing.

### IL-17 axis inhibitors

Vunakizumab (SHR-1314), an anti-IL-17A IgG1/κ antibody approved for the treatment of ankylosing spondylitis and psoriasis, was evaluated in a TED trial but was discontinued early for commercial reasons (140).

Another randomized, placebo-controlled, double-blind, parallel-group, multicenter interventional study involving patients with clinically active, moderate-to-severe TED (NCT04737330) investigated secukinumab, a recombinant, high-affinity, fully human monoclonal antibody that targets and blocks IL-17A. However, the findings of this *in vivo* study showed that secukinumab did not demonstrate clinical efficacy compared with placebo in the treatment of moderate-to-severe TED (24). Therefore, despite several findings suggesting an important involvement of IL-17A in TED, the results of this study may indicate that the Th17 axis is less dominant in the causal mechanism of TED than other mechanisms, such as those related to B-cell–driven autoimmunity (24). However, further studies are needed in larger cohorts of patients with TED.

### Combination and sequential therapies

Future therapeutic strategies may involve combining agents that target different aspects of the cytokine network or sequencing therapies to address distinct disease phases.

The clinical management of TED is particularly challenging due to its heterogeneity in presentation, unpredictable disease course, and variable response to treatment. Glucocorticoids continue to be recommended as first-line therapy; however, new therapies, including biologics and immunosuppressants, are emerging for use in steroid-refractory or steroid-intolerant cases (141).

Combining GCs with immunosuppressants has shown better clinical results than GCs alone (99, 102, 103, 106, 108). However, their efficacy is limited, and their use is restricted because of their toxicity profiles, including bone marrow suppression, nephrotoxicity, and hepatotoxicity (109).

The mainstay of medical management for GD is ATDs and betablockers; however, several new therapies are currently under investigation. The immunosuppressant azathioprine has also been investigated in GD combined with ATDs, showing promising results for TSH control, reducing TRAb levels, and lowering the relapse rate (107).

A randomized clinical trial in patients with GD evaluated the combined treatment of low-dose MTX to MMI, which resulted in a higher discontinuation rate and a faster decrease in TRAb levels than MMI alone (142, 143).

However, more studies are needed to evaluate the combination of different drugs in TED and GD patients who have different clinical profiles.

### Comparative evaluation of therapeutic agents in thyroid eye disease

The efficacy of high-dose intravenous GCs in the treatment of TED has been investigated in several studies with varying results. Responder patients have been reported with a prevalence ranging from approximately 51% to approximately 89%, with a mean responder rate across studies ranging between 70% and 80% (144, 145). GC treatment reduced CAS and inflammation in approximately 75% of patients but had only a slight effect on proptosis reduction (approximately –0,16 mm) (146), while improvement in diplopia was observed in approximately 19% of patients (146). A comparison of teprotumumab and corticosteroids reported in a recent paper (146) showed that teprotumumab is more effective than GCs in reducing proptosis (–2,31 mm) and improving diplopia.

Discordant results have been reported for rituximab. However, one control study showed that it was able to reduce CAS similarly to intravenous GCs, while another control study failed to demonstrate a significant effect of rituximab (89, 90​).

Recently, tocilizumab has been shown to reduce inflammation in patients who did not respond to intravenous GCs, reducing CAS (147).

## Biomarker-guided precision medicine

Targeted, mechanism-based therapies are transforming TED management, shifting treatment toward precision medicine. However, the identification of molecular markers to predict therapeutic response and guide the choice of biologics for personalized treatment is a critical and challenging issue (148). To date, TSI/TRAb are the most potent biomarkers available; with higher titers associated with an increased risk of more severe TED and TED relapse. However, the influence of high or rising TSI titers on surgical timing remains questionable (149, 150).

The role of the chemokines CXCL9 and CXCL10 as markers of TED activity and guides for therapeutic decision-making has been investigated in many studies (62–64). In particular, it has been shown that high levels of circulating CXCL10 are present in patients with active TED compared with those with inactive disease (62).

Furthermore, a significant reduction in CXCL10 and CXCL9 chemokines has been observed in patients with TED treated with corticosteroids and teleradiotherapy. The authors suggested that the concentrations of these Th1-dependent chemokines can reflect, at least in part, the activity of orbital inflammation and can be used to guide therapeutic decision-making in patients with TED (63).

A subsequent prospective study observed sustained decreases in serum CXCL10 levels in patients with TED after intravenous methylprednisolone treatment, and this decrease was associated with the clinical response to treatment. In contrast, other cytokines and chemokines (such as IL-4, IL-13, and Il-6) did not change after therapy, suggesting that CXCL10 can be used to evaluate the clinical response in these patients (64).

Furthermore, patients with newly diagnosed and relapsing GD have elevated CXCL10 levels, thus identifying the active phase of the disease (57). In GD, hyperthyroidism is associated with elevated Th1 chemokines, which are reduced by MMI, resulting in a shift toward a Th2 immune profile (58). Moreover, CXCL10 levels decrease after radioiodine ablation or thyroidectomy, indicating that the thyroid is the main source (59). CXCL10 polymorphisms are associated with disease severity, and persistently high levels may predict a lack of remission (60, 61).

A recent study investigated the metabolic profile of untreated patients with GD, demonstrating dysregulation within the metabolism of lipid and organic acid and identifying some metabolites such as diaminopimelic acid, N-phenethylacetamide, and Gly-Val as promising serum biomarkers for the diagnosis of GD (151).

Another study investigated a hypoxia biomarker-based diagnostic model for TED. Machine-learning algorithms identified critical hypoxia-related TED diagnostic genes that were associated with elevated TRAb levels, abnormal fT4, and higher infiltration by activated CD4+T cells and natural killer cells in patients with TED (152).

Recently, circulating levels of mediators involved in the fibrotic and inflammatory processes of TED, such as plasminogen activator inhibitor- 1 (PAI-1), TGF-*β*, and IGF-1R, were investigated in patients with different severities of TED, in patients with GD without TED, and in controls. PAI-1 appeared to have potential prognostic relevance for TED therapeutic stratification; reduced TGF-*β* levels were associated with clinical improvement, whereas elevated post-treatment PAI-1 levels were related to refractory diplopia, indicating a possible role in monitoring fibrotic extraocular muscle remodeling (153).

The role of extracellular vesicles (EVs) in immune regulation and tissue remodeling in autoimmune diseases has recently emerged. EVs have been shown to be actively involved in GD and TED, indicating their possible role as diagnostic and prognostic markers (154, 155).

A retrospective study identified 25-hydroxy vitamin D as an independent predictor of intravenous GC response in TED. An increase of 25-hydroxy vitamin D during therapy may be associated with improved treatment outcomes, particularly in male, elderly, and vitamin D-deficient patients (156).

A recent study measured serum levels of free fatty acids in patients with TED, demonstrating reduced levels of anti-inflammatory fatty acids. The authors showed that the eicosapentaenoic acid/arachidonic acid ratio may represent a solid, independent biomarker for TED activity, clinical assessment, and treatment selection (157).

Tear samples from patients with TED have also undergone lipidomic analysis. Among all lipids investigated, four were selected, showing good predictive ability for TED activity. These lipids were significantly correlated with blood lipid indicators, the degree of exophthalmos, the Schirmer I test, and the area of the foveal avascular zone of the retina (158).

## Future research directions

Current treatment of GD emphasizes thyroid-preserving strategies with long-term ATDs therapy. Future therapies will be increasingly focused on the immunological mechanisms of the disease; novel immunotherapies targeting the TRAb life cycle, such as those aimed at reducing their production, enhancing their clearance, or impairing their target, are under clinical evaluation (137).

The therapeutic landscape of TED is also rapidly evolving with advances in the understanding of autoimmune mechanisms and the emergence of targeted biologics that have reshaped treatment strategies. Whereas corticosteroids primarily reduce inflammation, teprotumumab effectively improves proptosis and diplopia, tocilizumab reduces both inflammation and proptosis, and rituximab has been associated with early disease inactivation and reduced relapse rates (148, 159).

The ETA/ATA consensus on the management of TED (160) suggests choosing therapy based on the efficacy of a specific drug on the dominant clinical feature of the disease in an individual patient, rather than following a first-line/second-line approach. This strategy may gain importance as emerging therapies advance through clinical trials and combination regimens are considered (159).

Extended follow-up studies of currently investigated therapies may help define the durability of response and late treatment-related events and evaluate combined treatments or the addition of short courses of corticosteroids during acute phases (148).

Another issue that must be addressed is the clinical heterogeneity of TED, including variability across racial and ethnic groups. For example, patients of Asian ancestry more frequently exhibit difference in CAS and proptosis that can influence risk stratification, diagnostic sensitivity, and treatment selection.

Moreover, despite the availability of new emerging therapies, 20%–30% of patients with TED still require additional surgical intervention due to an inadequate or partial response to optimal medical management.

Future research may provide a deeper understanding of TED pathogenesis, including conjunctival vascular alterations and multiomics–based phenotyping integrating racial and anatomical factors, thereby enabling more personalized treatment strategies (161).

Furthermore, another critical point is access to new biological treatments, which remains unaffordable and inconsistent in many global regions. Lower-cost IGF-1R biosimilars are emerging in some countries, including China. If biologics are unavailable, steroids, conventional immunosuppressants, radiotherapy, and surgery remain effective options. International guidelines should take into account local realities and validated non-biologic pathways (149).

In the future, new technology based on artificial intelligence will enhance the diagnosis, treatment, and monitoring of these diseases. Currently, deep-learning models have been shown to differentiate GD from thyroiditis, predict relapse after ATDs withdrawal, and assess disease severity and treatment response in TED. Although this technological advancement may enable more personalized care, its clinical utility remains limited because of the lack of prospective validation, data standardization, and regulatory approval (137).

However, the best treatment remains prevention whenever possible, including smoking cessation and early recognition of disease. Additional research is needed to determine whether optimizing vitamin D levels, reducing exposure to air pollution and endocrine disruptors, managing stress, and adhering to a Mediterranean dietary pattern can provide protective benefits (149).

## Conclusions

The role of cytokines is central in the inflammatory process of GD and TED, and drugs targeting cytokines allow a more precise intervention than traditional therapies, which primarily provide symptomatic control. Cytokine-targeted therapies have revolutionized TED management, whereas their role in GD remains limited. Nevertheless, new insights obtained from TED research may aid in future strategies to prevent extrathyroidal manifestations or modify the disease course. Conventional therapies may be combined with new therapies aimed at targeting upstream immune pathways regulating cytokine networks, such as teprotumumab, thereby providing substantial clinical benefits. However, several questions remain unanswered, such as the identification of optimal treatment windows for different therapeutic targets, whether biomarkers can guide treatment selection, how long-term safety can be ensured with chronic or repeated biologic therapy, and whether combined therapy can improve outcomes without unacceptable toxicity. Overall, the management of both GD and TED requires multidisciplinary collaboration among specialists in endocrinology, immunology, and ophthalmology, and new advancements in the management of these disorders will likely arise from ongoing research into cytokine biology, therapeutic targets, and patient stratification.

## References

[1] Antonelli A Ferrari SM Corrado A Di Domenicantonio A Fallahi P . Autoimmune thyroid disorders. Autoimmun Rev. (2015) 14:174–80. doi: 10.1016/j.autrev.2014.10.016 25461470

[2] Vargas-Uricoechea H Castellanos-Pinedo A Urrego-Noguera K Pinzón-Fernández MV Meza-Cabrera IA Vargas-Sierra H . A scoping review on the prevalence of Hashimoto's thyroiditis and the possible associated factors. Med Sci (Basel). (2025) 13:43. doi: 10.3390/medsci13020043 40265390 PMC12015930

[3] Ferrari SM Fallahi P Elia G Ragusa F Camastra S Paparo SR . Novel therapies for thyroid autoimmune diseases: An update. Best Pract Res Clin Endocrinol Metab. (2020) 34:101366. doi: 10.1016/j.beem.2019.101366 31813786

[4] Vaidya B Pearce SHS . Diagnosis and management of thyrotoxicosis. BMJ. (2014) 349:g5128–8. doi: 10.1136/bmj.g5128 25146390

[5] Sm M B R . Breaking tolerance to thyroid antigens: changing concepts in thyroid autoimmunity. Endocr Rev. (2014) 35:59–105. doi: 10.1210/er.2013-1055 24091783 PMC3895862

[6] Kotwal A Stan M . Thyrotropin receptor antibodies-an overview. Ophthal Plast Reconstr Surg. (2018) 34:S20–7. doi: 10.1097/IOP.0000000000001052 29771756

[7] Diana T Olivo PD Kahaly GJ . Thyrotropin receptor blocking antibodies. Horm Metab Res Horm Stoffwechselforschung Horm Metab. (2018) 50:853–62. doi: 10.1055/a-0723-9023 30286485 PMC6290727

[8] Antonelli A Fallahi P Elia G Ragusa F Paparo SR Ruffilli I . Graves’ disease: Clinical manifestations, immune pathogenesis (cytokines and chemokines) and therapy. Best Pract Res Clin Endocrinol Metab. (2020) 34:101388. doi: 10.1016/j.beem.2020.101388 32059832

[9] Smith TJ Hegedüs L . Graves’ disease. N Engl J Med. (2016) 375:1552–65. doi: 10.1056/NEJMra1510030 27797318

[10] Stein JD Childers D Gupta S Talwar N Nan B Lee BJ . Risk factors for developing thyroid-associated ophthalmopathy among individuals with Graves disease. JAMA Ophthalmol. (2015) 133:290–6. doi: 10.1001/jamaophthalmol.2014.5103 25502604 PMC4495733

[11] Yoon JS Kikkawa DO . Thyroid eye disease: From pathogenesis to targeted therapies. Taiwan J Ophthalmol. (2022) 12:3–11. doi: 10.4103/tjo.tjo_51_21 35399971 PMC8988977

[12] Xia N Zhou S Liang Y Xiao C Shen H Pan H . CD4+ T cells and the Th1/Th2 imbalance are implicated in the pathogenesis of Graves’ ophthalmopathy. Int J Mol Med. (2006) 17:911–6. doi: 10.3892/ijmm.17.5.911 16596280

[13] Aniszewski JP Valyasevi RW Bahn RS . Relationship between disease duration and predominant orbital T cell subset in Graves’ ophthalmopathy. J Clin Endocrinol Metab. (2000) 85:776–80. doi: 10.1210/jcem.85.2.6333 10690890

[14] Crescioli C Cosmi L Borgogni E Santarlasci V Gelmini S Sottili M . Methimazole inhibits CXC chemokine ligand 10 secretion in human thyrocytes. J Endocrinol. (2007) 195:145–55. doi: 10.1677/JOE-07-0240 17911406

[15] Wakelkamp IMMJ Bakker O Baldeschi L Wiersinga WM Prummel MF . TSH-R expression and cytokine profile in orbital tissue of active vs. inactive Graves’ ophthalmopathy patients. Clin Endocrinol (Oxf). (2003) 58:280–7. doi: 10.1046/j.1365-2265.2003.01708.x 12608932

[16] Viola N Colleo A Casula M Mura C Boi F Lanzolla G . Graves’ disease: Is it time for targeted therapy? A narrative review. Med (Mex). (2025) 61:500. doi: 10.3390/medicina61030500 40142311 PMC11943693

[17] Yang Y Hui C . Monoclonal antibodies to thyrotropin receptor with thyroid-stimulating activity activate the NF-κB pathway to induce chemokine expression. J Cell Mol Med. (2025) 29:e70647. doi: 10.1111/jcmm.70647 40500868 PMC12158658

[18] Ferrari SM Ruffilli I Elia G Ragusa F Paparo SR Patrizio A . Chemokines in hyperthyroidism. J Clin Transl Endocrinol. (2019) 16:100196. doi: 10.1016/j.jcte.2019.100196 31193493 PMC6536457

[19] Kristensen B . Regulatory B and T cell responses in patients with autoimmune thyroid disease and healthy controls. Dan Med J. (2016) 63:B5177. 26836805

[20] Weaver CT Hatton RD Mangan PR Harrington LE . IL-17 family cytokines and the expanding diversity of effector T cell lineages. Annu Rev Immunol. (2007) 25:821–52. doi: 10.1146/annurev.immunol.25.022106.141557 17201677

[21] Ruggeri RM Saitta S Cristani M Giovinazzo S Tigano V Trimarchi F . Serum interleukin-23 (IL-23) is increased in Hashimoto’s thyroiditis. Endocr J. (2014) 61:359–63. doi: 10.1507/endocrj.ej13-0484 24476945

[22] Li D Cai W Gu R Zhang Y Zhang H Tang K . Th17 cell plays a role in the pathogenesis of Hashimoto’s thyroiditis in patients. Clin Immunol. (2013) 149:411–20. doi: 10.1016/j.clim.2013.10.001 24211715

[23] Fang J Yu L Zhuang L-G Pei X-Y Wang Q Jin G-X . The changes in peripheral blood Th17 and Treg ratios in Hashimoto’s thyroiditis are accompanied by differential PD-1/PD-L1 expression. Front Endocrinol. (2022) 13:959477. doi: 10.3389/fendo.2022.959477 36093111 PMC9454193

[24] Wolf J Lorenz K Othman AE Beck A Michel HM Grauhan L . Secukinumab in moderate-to-severe Graves’ orbitopathy: A randomized, double-blind, placebo-controlled, multicenter study. J Clin Endocrinol Metab. (2025) 111:dgaf655. doi: 10.1210/clinem/dgaf655 41362233 PMC13099189

[25] Santaguida MG Gatto I Mangino G Virili C Stramazzo I Fallahi P . BREG cells in Hashimoto's thyroiditis isolated or associated to further organ-specific autoimmune diseases. Clin Immunol. (2017) 184:42–7. doi: 10.1016/j.clim.2017.04.012 28461108

[26] Ferrari SM Fallahi P Galdiero MR Ruffilli I Elia G Ragusa F . Immune and inflammatory cells in thyroid cancer microenvironment. Int J Mol Sci. (2019) 20:4413. doi: 10.3390/ijms20184413 31500315 PMC6769504

[27] Ferrari SM Paparo SR Ragusa F Elia G Mazzi V Patrizio A . Chemokines in thyroid autoimmunity. Best Pract Res Clin Endocrinol Metab. (2023) 37:101773. doi: 10.1016/j.beem.2023.101773 36907786

[28] Elia G Fallahi P Ragusa F Paparo SR Mazzi V Benvenga S . Precision medicine in Graves’ disease and ophthalmopathy. Front Pharmacol. (2021) 12:754386. doi: 10.3389/fphar.2021.754386 34776972 PMC8581657

[29] Ciarmatori N Quaranta Leoni F Quaranta Leoni FM . Redefining treatment paradigms in thyroid eye disease: Current and future therapeutic strategies. J Clin Med. (2025) 14:5528. doi: 10.3390/jcm14155528 40807149 PMC12347536

[30] Guo Q Wu Y Hou Y Liu Y Liu T Zhang H . Cytokine secretion and pyroptosis of thyroid follicular cells mediated by enhanced NLRP3, NLRP1, NLRC4, and AIM2 inflammasomes are associated with autoimmune thyroiditis. Front Immunol. (2018) 9:1197. doi: 10.3389/fimmu.2018.01197 29915579 PMC5994487

[31] Neamțu M Bild V Vasincu A Arcan OD Bulea D Ababei D-C . Inflammasome molecular insights in autoimmune diseases. Curr Issues Mol Biol. (2024) 46:3502–32. doi: 10.3390/cimb46040220 38666950 PMC11048795

[32] Liu N Li X Liu C Zhao Y Cui B Ning G . The association of interleukin-1alpha and interleukin-1beta polymorphisms with the risk of Graves’ disease in a case-control study and meta-analysis. Hum Immunol. (2010) 71:397–401. doi: 10.1016/j.humimm.2010.01.023 20116409

[33] Li B Smith TJ . Divergent expression of IL-1 receptor antagonists in CD34⁺ fibrocytes and orbital fibroblasts in thyroid-associated ophthalmopathy: contribution of fibrocytes to orbital inflammation. J Clin Endocrinol Metab. (2013) 98:2783–90. doi: 10.1210/jc.2013-1245 23633206 PMC3701275

[34] Aoun T Danielova Gueorguieva D Wu KY . Orbital inflammation in thyroid eye disease: Stress responses and their implications. Stresses. (2024) 4:54–78. doi: 10.3390/stresses4010004 30654563

[35] Pol JG Caudana P Paillet J Piaggio E Kroemer G . Effects of interleukin-2 in immunostimulation and immunosuppression. J Exp Med. (2020) 217:e20191247. doi: 10.1084/jem.20191247 31611250 PMC7037245

[36] Naka T Nishimoto N Kishimoto T . The paradigm of IL-6: from basic science to medicine. Arthritis Res. (2002) 4:S233–42. doi: 10.1186/ar565 12110143 PMC3240141

[37] Murdock J Nguyen J Hurtgen BJ Andorfer C Walsh J Lin A . The role of IL-6 in thyroid eye disease: an update on emerging treatments. Front Ophthalmol. (2025) 5:1544436. doi: 10.3389/fopht.2025.1544436 40297767 PMC12034681

[38] Tanaka T Narazaki M Kishimoto T . Interleukin (IL-6) immunotherapy. Cold Spring Harb Perspect Biol. (2018) 10:a028456. doi: 10.1101/cshperspect.a028456 28778870 PMC6071487

[39] He H Jiang Y Qiu J Shen F Qian D Meng L . Role of interleukin 17 and T helper cells 17 cells as a new immune target and signalling in the pathogenesis and treatment of autoimmune thyroid diseases. Ann Med. (2025) 57:2586216. doi: 10.1080/07853890.2025.2586216 41243116 PMC12624951

[40] Afzali B Lombardi G Lechler RI Lord GM . The role of T helper 17 (Th17) and regulatory T cells (Treg) in human organ transplantation and autoimmune disease. Clin Exp Immunol. (2007) 148:32–46. doi: 10.1111/j.1365-2249.2007.03356.x 17328715 PMC1868863

[41] Kościuszko M Popławska-Kita A Pawłowski P Lipińska D Hryniewicka J Jankowska D . Clinical relevance of estimating circulating interleukin-17 and interleukin-23 during methylprednisolone therapy in Graves’ orbitopathy: A preliminary study. Adv Med Sci. (2021) 66:315–20. doi: 10.1016/j.advms.2021.07.002 34256242

[42] Zhang P Zhu H . Cytokines in thyroid-associated ophthalmopathy. J Immunol Res. (2022) 2022:2528046. doi: 10.1155/2022/2528046 36419958 PMC9678454

[43] Fallahi P Ferrari SM Elia G Ragusa F Paparo SR Patrizio A . Cytokines as targets of novel therapies for Graves’ ophthalmopathy. Front Endocrinol. (2021) 12:654473. doi: 10.3389/fendo.2021.654473 33935970 PMC8085526

[44] Zlotnik A Yoshie O . Chemokines: a new classification system and their role in immunity. Immunity. (2000) 12:121–7. doi: 10.1016/s1074-7613(00)80165-x 10714678

[45] Arenberg DA Polverini PJ Kunkel SL Shanafelt A Hesselgesser J Horuk R . The role of CXC chemokines in the regulation of angiogenesis in non-small cell lung cancer. J Leukoc Biol. (1997) 62:554–62. doi: 10.1002/jlb.62.5.554 9365108

[46] Rotondi M Lazzeri E Romagnani P Serio M . Role for interferon-gamma inducible chemokines in endocrine autoimmunity: an expanding field. J Endocrinol Invest. (2003) 26:177–80. doi: 10.1007/BF03345149 12739748

[47] Antonelli A Fallahi P Rotondi M Ferrari SM Romagnani P Grosso M . Increased serum CXCL10 in Graves’ disease or autoimmune thyroiditis is not associated with hyper- or hypothyroidism per se, but is specifically sustained by the autoimmune, inflammatory process. Eur J Endocrinol. (2006) 154:651–8. doi: 10.1530/eje.1.02137 16645011

[48] Antonelli A Ferrari SM Giuggioli D Ferrannini E Ferri C Fallahi P . Chemokine (C-X-C motif) ligand (CXCL)10 in autoimmune diseases. Autoimmun Rev. (2014) 13:272–80. doi: 10.1016/j.autrev.2013.10.010 24189283

[49] Romagnani P Rotondi M Lazzeri E Lasagni L Francalanci M Buonamano A . Expression of IP-10/CXCL10 and MIG/CXCL9 in the thyroid and increased levels of IP-10/CXCL10 in the serum of patients with recent-onset Graves’ disease. Am J Pathol. (2002) 161:195–206. doi: 10.1016/S0002-9440(10)64171-5 12107104 PMC1850693

[50] Kemp EH Metcalfe RA Smith KA Woodroofe MN Watson PF Weetman AP . Detection and localization of chemokine gene expression in autoimmune thyroid disease. Clin Endocrinol (Oxf). (2003) 59:207–13. doi: 10.1046/j.1365-2265.2003.01824.x 12864798

[51] Antonelli A Rotondi M Fallahi P Romagnani P Ferrari SM Paolicchi A . Increase of interferon-gamma inducible alpha chemokine CXCL10 but not beta chemokine CCL2 serum levels in chronic autoimmune thyroiditis. Eur J Endocrinol. (2005) 152:171–7. doi: 10.1530/eje.1.01847 15745922

[52] García-López MA Sancho D Sánchez-Madrid F Marazuela M . Thyrocytes from autoimmune thyroid disorders produce the chemokines IP-10 and Mig and attract CXCR3+ lymphocytes. J Clin Endocrinol Metab. (2001) 86:5008–16. doi: 10.1210/jcem.86.10.7953 11600578

[53] Romagnani P Annunziato F Lasagni L Lazzeri E Beltrame C Francalanci M . Cell cycle-dependent expression of CXC chemokine receptor 3 by endothelial cells mediates angiostatic activity. J Clin Invest. (2001) 107:53–63. doi: 10.1172/JCI9775 11134180 PMC198541

[54] Aust G Sittig D Steinert M Lamesch P Lohmann T . Graves’ disease is associated with an altered CXCR3 and CCR5 expression in thyroid-derived compared to peripheral blood lymphocytes. Clin Exp Immunol. (2002) 127:479–85. doi: 10.1046/j.1365-2249.2002.01778.x 11966764 PMC1906316

[55] Antonelli A Rotondi M Fallahi P Romagnani P Ferrari SM Buonamano A . High levels of circulating CXC chemokine ligand 10 are associated with chronic autoimmune thyroiditis and hypothyroidism. J Clin Endocrinol Metab. (2004) 89:5496–9. doi: 10.1210/jc.2004-0977 15531503

[56] Ferrari SM Ragusa F Paparo SR Nasini F Nardi M Franceschini SS . Differential modulation of CXCL8 versus CXCL10, by cytokines, PPAR-gamma, or PPAR-alpha agonists, in primary cells from Graves’ disease and ophthalmopathy. Autoimmun Rev. (2019) 18:673–8. doi: 10.1016/j.autrev.2019.05.004 31059842

[57] Antonelli A Rotondi M Fallahi P Romagnani P Ferrari SM Barani L . Increase of interferon-gamma-inducible CXC chemokine CXCL10 serum levels in patients with active Graves’ disease, and modulation by methimazole therapy. Clin Endocrinol (Oxf). (2006) 64:189–95. doi: 10.1111/j.1365-2265.2006.02447.x 16430719

[58] Inukai Y Momobayashi A Sugawara N Aso Y . Changes in expression of T-helper (Th) 1- and Th2-associated chemokine receptors on peripheral blood lymphocytes and plasma concentrations of their ligands, interferon-inducible protein-10 and thymus and activation-regulated chemokine, after antithyroid drug administration in hyperthyroid patients with Graves’ disease. Eur J Endocrinol. (2007) 156:623–30. doi: 10.1530/EJE-07-0019 17535861

[59] Antonelli A Rotondi M Fallahi P Grosso M Boni G Ferrari SM . Iodine-131 given for therapeutic purposes modulates differently interferon-gamma-inducible alpha-chemokine CXCL10 serum levels in patients with active Graves’ disease or toxic nodular goiter. J Clin Endocrinol Metab. (2007) 92:1485–90. doi: 10.1210/jc.2006-1571 17244787

[60] Brück P Bartsch W Sadet D Penna-Martinez M Kurylowicz A Bednarczuk T . A CXC motif ligand 10 polymorphism as a marker to predict severity of Graves’ disease. Thyroid Off J Am Thyroid Assoc. (2010) 20:343–5. doi: 10.1089/thy.2009.0222 20187787

[61] Sakai H Togawa Y Fukuda G Ito R Miwa T Odawara M . Serum chemokine (C-X-C motif) ligand 10 levels are elevated in patients with Graves’ disease in long-term remission. Thyroid Off J Am Thyroid Assoc. (2010) 20:341–2. doi: 10.1089/thy.2009.0179 20144041

[62] Antonelli A Rotondi M Ferrari SM Fallahi P Romagnani P Franceschini SS . Interferon-gamma-inducible alpha-chemokine CXCL10 involvement in Graves’ ophthalmopathy: modulation by peroxisome proliferator-activated receptor-gamma agonists. J Clin Endocrinol Metab. (2006) 91:614–20. doi: 10.1210/jc.2005-1689 16303841

[63] Mysliwiec J Palyga I Kosciuszko M Kowalska A Gorska M . Circulating CXCL9 and CXCL10 as markers of activity of Graves’ orbitopathy during treatment with corticosteroids and teleradiotherapy. Horm Metab Res Horm Stoffwechselforschung Horm Metab. (2012) 44:957–61. doi: 10.1055/s-0032-1316352 22752955

[64] Zhu W Ye L Shen L Jiao Q Huang F Han R . A prospective, randomized trial of intravenous glucocorticoids therapy with different protocols for patients with graves’ ophthalmopathy. J Clin Endocrinol Metab. (2014) 99:1999–2007. doi: 10.1210/jc.2013-3919 24606088

[65] Appay V Rowland-Jones SL . RANTES: a versatile and controversial chemokine. Trends Immunol. (2001) 22:83–7. doi: 10.1016/s1471-4906(00)01812-3 11286708

[66] Aldinucci D Gloghini A Pinto A Colombatti A Carbone A . The role of CD40/CD40L and interferon regulatory factor 4 in Hodgkin lymphoma microenvironment. Leuk Lymphoma. (2012) 53:195–201. doi: 10.3109/10428194.2011.605190 21756027

[67] Wan S Lin M Mao Y Chen X Liang D . Altered expression of CXCL13 and its chemokine receptor CXCR5 on B lymphocytes during active Graves’ orbitopathy. Curr Eye Res. (2021) 46:210–6. doi: 10.1080/02713683.2020.1786132 32643429

[68] Cain DW Cidlowski JA . Immune regulation by glucocorticoids. Nat Rev Immunol. (2017) 17:233–47. doi: 10.1038/nri.2017.1 28192415 PMC9761406

[69] Längericht J Krämer I Kahaly GJ . Glucocorticoids in Graves’ orbitopathy: mechanisms of action and clinical application. Ther Adv Endocrinol Metab. (2020) 11:2042018820958335. doi: 10.1177/2042018820958335 33403097 PMC7745544

[70] Duarte AF Xavier NF Sales Sanz M Cruz AAV . Efficiency and safety of tocilizumab for the treatment of thyroid eye disease: a systematic review. Ophthal Plast Reconstr Surg. (2024) 40:367–73. doi: 10.1097/IOP.0000000000002573 38215463

[71] Sun A Wang X Qu J Wu Y . The efficacy and safety of intravenous tocilizumab to treat Graves’ ophthalmopathy: a systematic review and single-arm meta-analysis. J Clin Endocrinol Metab. (2025) 110:e886–96. doi: 10.1210/clinem/dgae711 39401327

[72] Lamb YN Deeks ED . Sarilumab: a review in moderate to severe rheumatoid arthritis. Drugs. (2018) 78:929–40. doi: 10.1007/s40265-018-0929-z 29931592

[73] Moreira Navarrete V Toyos Sáenz de Miera FJ Garrido Hermosilla AM Muñoz Reinoso P Madrigal Domínguez MJ Pérez Venegas JJ . Scientific abstract. AB1315 Sarilumab in patients with refractory Graves’ orbitopathy. Effectiveness and safety in a series of cases in clinical practice. Ann Rheum Dis. (2022) 81:1764–5. doi: 10.1136/annrheumdis-2022-eular.4039

[74] Pérez-Moreiras J Abelenda D Providência J Castela G . Sarilumab in the management of Graves orbitopathy with low clinical activity scores. Sci Rep. (2026) 16:5225. doi: 10.1038/s41598-026-35682-4 41535480 PMC12881573

[75] Heo Y-A . Satralizumab: first approval. Drugs. (2020) 80:1477–82. doi: 10.1007/s40265-020-01380-2 32797372 PMC7522096

[76] ENSPRYNG (satralizumab-mwge) injection. In: Highlights of Prescribing Information. Available online at: https://www.gene.com/download/pdf/enspryng_prescribing.pdf (Accessed June 15, 2026).

[77] Ezra D Collins A Haskova Z Kuenzel T Ida H Triyatni M . Targeting IL-6 receptor signaling with satralizumab in thyroid eye disease: design of the phase 3 SatraGO-1 and SatraGO-2 trials. Ophthalmol Ther. (2025) 14:3119–32. doi: 10.1007/s40123-025-01255-3 41066062 PMC12579074

[78] Boskovic O Medenica S Radojevic N Zarkovic M . Etanercept in the treatment of Graves' ophthalmopathy with primary hypothyroidism and rheumatoid arthritis. Cent Eur J Immunol. (2019) 44:463–5. doi: 10.5114/ceji.2019.92803 32140060 PMC7050053

[79] Paridaens D van den Bosch WA van der Loos TL Krenning EP van Hagen PM . The effect of etanercept on Graves’ ophthalmopathy: a pilot study. Eye. (2005) 19:1286–9. doi: 10.1038/sj.eye.6701768 15550932

[80] Durrani OM Reuser TQ Murray PI . Infliximab: a novel treatment for sight-threatening thyroid associated ophthalmopathy. Orbit. (2005) 24:117–9. doi: 10.1080/01676830590912562 16191800

[81] Komorowski J Jankiewicz-Wika J Siejka A Lawnicka H Kłysik A Goś R . Monoclonal anti-TNFalpha antibody (infliximab) in the treatment of patient with thyroid associated ophthalmopathy. Klin Oczna. (2007) 109:457–60. 18488396

[82] Ayabe R Rootman DB Hwang CJ Ben-Artzi A Goldberg R . Adalimumab as steroid-sparing treatment of inflammatory-stage thyroid eye disease. Ophthal Plast Reconstr Surg. (2014) 30:415–9. doi: 10.1097/IOP.0000000000000211 24978425

[83] El Fassi D Banga JP Gilbert JA Padoa C Hegedüs L Nielsen CH . Treatment of Graves’ disease with rituximab specifically reduces the production of thyroid stimulating autoantibodies. Clin Immunol. (2009) 130:252–8. doi: 10.1016/j.clim.2008.09.007 18964302

[84] Heemstra KA Toes RE Sepers J Pereira AM Corssmit EP Huizinga TWJ . Rituximab in relapsing Graves’ disease, a phase II study. Eur J Endocrinol. (2008) 159:609–15. doi: 10.1530/EJE-08-0084 18628345

[85] Cheetham TD Cole M Abinun M Allahabadia A Barratt T Davies JH . Adjuvant Rituximab-Exploratory Trial in Young People With Graves Disease. J Clin Endocrinol Metab. (2022) 107:743–54. doi: 10.1210/clinem/dgab763 34687316 PMC8851941

[86] El Fassi D Nielsen CH Bonnema SJ Hasselbalch HC Hegedüs L . B lymphocyte depletion with the monoclonal antibody rituximab in Graves’ disease: a controlled pilot study. J Clin Endocrinol Metab. (2007) 92:1769–72. doi: 10.1210/jc.2006-2388 17284622

[87] El Fassi D Nielsen CH Hasselbalch HC Hegedüs L . The rationale for B lymphocyte depletion in Graves’ disease. Monoclonal anti-CD20 antibody therapy as a novel treatment option. Eur J Endocrinol. (2006) 154:623–32. doi: 10.1530/eje.1.02140 16645007

[88] Mitchell AL Gan EH Morris M Johnson K Neoh C Dickinson AJ . The effect of B cell depletion therapy on anti-TSH receptor antibodies and clinical outcome in glucocorticoid-refractory Graves’ orbitopathy. Clin Endocrinol (Oxf). (2013) 79:437–42. doi: 10.1111/cen.12141 23320840

[89] Salvi M Vannucchi G Currò N Campi I Covelli D Dazzi D . Efficacy of B-cell targeted therapy with rituximab in patients with active moderate to severe Graves’ orbitopathy: a randomized controlled study. J Clin Endocrinol Metab. (2015) 100:422–31. doi: 10.1210/jc.2014-3014 25494967 PMC4318899

[90] Stan MN Garrity JA Carranza Leon BG Prabin T Bradley EA Bahn RS . Randomized controlled trial of rituximab in patients with Graves’ orbitopathy. J Clin Endocrinol Metab. (2015) 100:432–41. doi: 10.1210/jc.2014-2572 25343233 PMC4318907

[91] Du Pasquier-Fediaevsky L Andrei S Berche M Leenhardt L Héron E Rivière S . Low-dose rituximab for active moderate to severe Graves’ orbitopathy resistant to conventional treatment. Ocul Immunol Inflammation. (2019) 27:844–50. doi: 10.1080/09273948.2018.1453078 29652204

[92] Insull EA Sipkova Z David J Turner HE Norris JH . Early low-dose rituximab for active thyroid eye disease: an effective and well-tolerated treatment. Clin Endocrinol (Oxf). (2019) 91:179–86. doi: 10.1111/cen.13970 30864162

[93] Shen W-C Lee C-H Loh E-W Hsieh A-T Chen L Tam K-W . Efficacy and safety of rituximab for the treatment of Graves’ orbitopathy: a meta-analysis of randomized controlled trials. Pharmacotherapy. (2018) 38:503–10. doi: 10.1002/phar.2111 29601105

[94] El Fassi D Nielsen CH Junker P Hasselbalch HC Hegedüs L . Systemic adverse events following rituximab therapy in patients with Graves’ disease. J Endocrinol Invest. (2011) 34:e163–7. doi: 10.3275/7411 21169731

[95] Bartalena L Kahaly GJ Baldeschi L Dayan CM Eckstein A Marcocci C . The 2021 European Group on Graves’ orbitopathy (EUGOGO) clinical practice guidelines for the medical management of Graves’ orbitopathy. Eur J Endocrinol. (2021) 185:G43–67. doi: 10.1530/EJE-21-0479 34297684

[96] Ghahvehchian H Eshraghi B . Tofacitinib for refractory thyroid eye disease. JAMA Ophthalmol. (2025) 143:1073–5. doi: 10.1001/jamaophthalmol.2025.3974 41165674

[97] Dasgupta A . Therapeutic drug monitoring of mycophenolic acid. Adv Clin Chem. (2016) 76:165–84. doi: 10.1016/bs.acc.2016.04.001 27645819

[98] Zwerner J Fiorentino D . Mycophenolate mofetil. Dermatol Ther. (2007) 20:229–38. doi: 10.1111/j.1529-8019.2007.00136.x 17970888

[99] Li L-F Xue J-L Guan L Su F-F Wang H Zhang D-F . Therapeutic outcomes of mycophenolate mofetil and glucocorticoid in thyroid-associated ophthalmopathy patients. Front Endocrinol. (2023) 14:1140196. doi: 10.3389/fendo.2023.1140196 37025403 PMC10070944

[100] Quah Qin Xian N Alnahrawy A Akshikar R Lee V . Real-world efficacy and safety of mycophenolate mofetil in active moderate-to-sight-threatening thyroid eye disease. Clin Ophthalmol. (2021) 15:1921–32. doi: 10.2147/OPTH.S305717 34007144 PMC8121682

[101] Witowska A Pawluczuk E Szczepanek-Parulska E Ruchala M . The role of mycophenolate mofetil in the therapy of thyroid eye disease. Ther Adv Endocrinol Metab. (2025) 16:20420188251383022. doi: 10.1177/20420188251383022 41180578 PMC12575950

[102] Zloto O Rosset A Priel A Landau-Prat D Cukierman-Yaffe T Shavit R . Elevated serum thyroid stimulating immunoglobulin linked to failure of first-line intravenous methylprednisolone monotherapy in moderate-to-severe thyroid eye disease. Eye. (2024) 38:687–90. doi: 10.1038/s41433-023-02748-w 37821543 PMC10920676

[103] Rajabi MT Rafizadeh SM Mohammadi A Eshraghi B Mohammadi N Hosseini SS . Mycophenolate mofetil (CellCept®) in combination with low dose prednisolone in moderate to severe Graves’ orbitopathy. Front Med. (2022) 9:788228. doi: 10.3389/fmed.2022.788228 35223896 PMC8873183

[104] Kauppinen-Mäkelin R Karma A Leinonen E Löyttyniemi E Salonen O Sane T . High dose intravenous methylprednisolone pulse therapy versus oral prednisone for thyroid-associated ophthalmopathy. Acta Ophthalmol Scand. (2002) 80:316–21. doi: 10.1034/j.1600-0420.2002.800316.x 12059873

[105] Perros P Weightman DR Crombie AL Kendall-Taylor P . Azathioprine in the treatment of thyroid-associated ophthalmopathy. Acta Endocrinol (Copenh). (1990) 122:8–12. doi: 10.1530/acta.0.1220008 2305608

[106] Rajendram R Taylor PN Wilson VJ Harris N Morris OC Tomlinson M . Combined immunosuppression and radiotherapy in thyroid eye disease (CIRTED): a multicentre, 2 × 2 factorial, double-blind, randomised controlled trial. Lancet Diabetes Endocrinol. (2018) 6:299–309. doi: 10.1016/S2213-8587(18)30021-4 29396245

[107] Allam MM El-Zawawy HT Kader Okda AA Ali Alshaikh A Ghazy RM . Azathioprine as an adjuvant therapy in severe Graves' disease: a randomized controlled open-label clinical trial. Front Endocrinol (Lausanne). (2023) 14:1168936. doi: 10.3389/fendo.2023.1168936 37409226 PMC10319122

[108] Prummel MF Mourits MP Berghout A Krenning EP van der Gaag R Koornneef L . Prednisone and cyclosporine in the treatment of severe Graves' ophthalmopathy. N Engl J Med. (1989) 321:1353–9. doi: 10.1056/NEJM198911163212002 2519530

[109] Strianese D Rossi F . Interruption of autoimmunity for thyroid eye disease: B-cell and T-cell strategy. Eye (Lond). (2019) 33:191–9. doi: 10.1038/s41433-018-0315-9 30610229 PMC6367473

[110] Arenas M Sabater S Jiménez PL Rovirosa À Biete A Linares V . Radiotherapy for Graves’ disease. The possible role of low-dose radiotherapy. Rep Pract Oncol Radiother J Gt Cancer Cent Poznan Pol Soc Radiat Oncol. (2016) 21:213–8. doi: 10.1016/j.rpor.2016.02.001 27601953 PMC5002018

[111] Bartalena L Burch HB Burman KD Kahaly GJ . A 2013 European survey of clinical practice patterns in the management of Graves’ disease. Clin Endocrinol (Oxf). (2016) 84:115–20. doi: 10.1111/cen.12688 25581877

[112] Burch HB Cooper DS . Management of Graves disease: a review. JAMA. (2015) 314:2544–54. doi: 10.1001/jama.2015.16535 26670972

[113] Kahaly GJ Stan MN Frommer L Gergely P Colin L Amer A . A novel anti-CD40 monoclonal antibody, iscalimab, for control of Graves hyperthyroidism-a proof-of-concept trial. J Clin Endocrinol Metab. (2020) 105:dgz013. doi: 10.1210/clinem/dgz013 31512728

[114] Levy RA Gonzalez-Rivera T Khamashta M Fox NL Jones-Leone A Rubin B . 10 years of belimumab experience: What have we learnt? Lupus. (2021) 30:1705–21. doi: 10.1177/09612033211028653 34238087 PMC8564244

[115] Lin J-D Wang Y-H Fang W-F Hsiao C-J Chagnaadorj A Lin Y-F . Serum BAFF and thyroid autoantibodies in autoimmune thyroid disease. Clin Chim Acta Int J Clin Chem. (2016) 462:96–102. doi: 10.1016/j.cca.2016.09.004 27616625

[116] Gilbert JA Kalled SL Moorhead J Hess DM Rennert P Li Z . Treatment of autoimmune hyperthyroidism in a murine model of Graves’ disease with tumor necrosis factor-family ligand inhibitors suggests a key role for B cell activating factor in disease pathology. Endocrinology. (2006) 147:4561–8. doi: 10.1210/en.2006-0507 16794009

[117] Jansson L Vrolix K Jahraus A Martin KF Wraith DC . Immunotherapy with apitopes blocks the immune response to TSH receptor in HLA-DR transgenic mice. Endocrinology. (2018) 159:3446–57. doi: 10.1210/en.2018-00306 30099489

[118] Pearce SHS Dayan C Wraith DC Barrell K Olive N Jansson L . Antigen-specific immunotherapy with thyrotropin receptor peptides in Graves’ hyperthyroidism: a phase I study. Thyroid. (2019) 29:1003–11. doi: 10.1089/thy.2019.0036 31194638 PMC6648194

[119] Markham A . Teprotumumab: First approval. Drugs. (2020) 80:509–12. doi: 10.1007/s40265-020-01287-y 32157641

[120] TEPEZZA® (TEPROTUMUMAB) RECEIVES APPROVAL IN JAPAN FOR THE TREATMENT OF ACTIVE THYROID EYE DISEASE | Amgen Inc. Available online at: https://investors.amgen.com/news-releases/news-release-details/tepezzar-teprotumumab-receives-approval-japan-treatment-active/ (Accessed June 15, 2026).

[121] MHRA approves teprotumumab as the first UK treatment for adults with moderate to severe Thyroid Eye Disease (TED). GOVUK. Available online at: https://www.gov.uk/government/news/mhra-approves-teprotumumab-as-the-first-uk-treatment-for-adults-with-moderate-to-severe-thyroid-eye-disease-ted (Accessed June 15, 2026).

[122] Tepezza | European Medicines Agency (EMA) (2025). Available online at: https://www.ema.europa.eu/en/medicines/human/EPAR/tepezza (Accessed June 15, 2026).

[123] Smith TJ Kahaly GJ Ezra DG Fleming JC Dailey RA Tang RA . Teprotumumab for thyroid-associated ophthalmopathy. N Engl J Med. (2017) 376:1748–61. doi: 10.1056/NEJMoa1614949 28467880 PMC5718164

[124] Douglas RS Kahaly GJ Patel A Sile S Thompson EHZ Perdok R . Teprotumumab for the treatment of active thyroid eye disease. N Engl J Med. (2020) 382:341–52. doi: 10.1056/NEJMoa1910434 31971679

[125] Douglas RS Kahaly GJ Ugradar S Elflein H Ponto KA Fowler BT . Teprotumumab efficacy, safety, and durability in longer-duration thyroid eye disease and re-treatment: OPTIC-X study. Ophthalmology. (2022) 129:438–49. doi: 10.1016/j.ophtha.2021.10.017 34688699

[126] Douglas RS Couch S Wester ST Fowler BT Liu CY Subramanian PS . Efficacy and safety of teprotumumab in patients with thyroid eye disease of long duration and low disease activity. J Clin Endocrinol Metab. (2023) 109:25–35. doi: 10.1210/clinem/dgad637 37925673 PMC10735297

[127] Ugradar S Parunakian E Zimmerman E Malkhasyan E Raika P Douglas RN . Clinical and radiologic predictors of response to teprotumumab: A 3D volumetric analysis of 35 patients. Ophthal Plast Reconstr Surg. (2025) 41:408–14. doi: 10.1097/IOP.0000000000002867 39714282

[128] Sears CM Wang Y Bailey LA Turbin R Subramanian PS Douglas R . Early efficacy of teprotumumab for the treatment of dysthyroid optic neuropathy: A multicenter study. Am J Ophthalmol Case Rep. (2021) 23:101111. doi: 10.1016/j.ajoc.2021.101111 34113737 PMC8170359

[129] Mulvihill MJ Cooke A Rosenfeld-Franklin M Buck E Foreman K Landfair D . Discovery of OSI-906: a selective and orally efficacious dual inhibitor of the IGF-1 receptor and insulin receptor. Future Med Chem. (2009) 1:1153–71. doi: 10.4155/fmc.09.89 21425998

[130] Kaplan R Zhao Y Tsai J Dickinson B Swanson T Foster K . Preclinical pharmacology, pharmacokinetics, and pharmacodynamics of veligrotug, a full antagonist antibody to the IGF-1 receptor in development for thyroid eye disease. mAbs. (2025) 17:2585616. doi: 10.1080/19420862.2025.2585616 41250603 PMC12629334

[131] Sling therapeutics. Available online at: https://www.slingtx.com/2025/01/14/sling-therapeutics-announces-positive-topline-results-from-phase-2b-3-lids-clinical-trial-of-oral-small-molecule-linsitinib-in-patients-with-thyroid-eye-disease/ (Accessed June 15, 2026).

[132] Evans M Sanders J Tagami T Sanders P Young S Roberts E . Monoclonal autoantibodies to the TSH receptor, one with stimulating activity and one with blocking activity, obtained from the same blood sample. Clin Endocrinol (Oxf). (2010) 73:404–12. doi: 10.1111/j.1365-2265.2010.03831.x 20550534

[133] Furmaniak J Sanders J Sanders P Li Y Rees Smith B . TSH receptor specific monoclonal autoantibody K1-70TM targeting of the TSH receptor in subjects with Graves' disease and Graves' orbitopathy-results from a phase I clinical trial. Clin Endocrinol (Oxf). (2022) 96:878–87. doi: 10.1111/cen.14681 35088429 PMC9305464

[134] Noh JY Watanabe N Ito K Tsuiki M Ishihara Y Tagami T . Safety, pharmacokinetics, and potential benefits of TSH-receptor-specific monoclonal autoantibody K1-70^TM^ in Japanese Graves' disease patients: results of a phase 1 trial. Endocr J. (2025) 72:897–909. doi: 10.1507/endocrj.EJ25-0043 40301067 PMC12340244

[135] Kahaly GJ Steiner L van der Lee MMC van Achterberg TAE Arends RJ Karstens WFJ . Thyrotropin receptor antagonism by a novel small molecule: Preclinical *in vitro* observations. Thyroid. (2023) 33:732–42. doi: 10.1089/thy.2022.0694 37016815

[136] Krieger CC Neumann S Sui X Templin JS Kapri T Demillo VG . Inhibition of TSH receptor expression by a cyclotriazadisulfonamide as a potential treatment of Graves hyperthyroidism. Endocrinology. (2025) 166:bqaf037. doi: 10.1210/endocr/bqaf037 39964853 PMC11879233

[137] Stan MN Toro-Tobon D . Graves' disease: a new era of pathophysiology-guided therapeutics. J Clin Endocrinol Metab. (2026) 111:1212–24. doi: 10.1210/clinem/dgag010 41530911

[138] Kamboj A Harrison AR Mokhtarzadeh A . Emerging therapies in the medical management of thyroid eye disease. Front Ophthalmol. (2023) 3:1295902. doi: 10.3389/fopht.2023.1295902 38983101 PMC11182121

[139] Kahaly GJ Dolman PJ Wolf J Giers BC Elflein HM Jain AP . Proof-of-concept and randomized, placebo-controlled trials of an FcRn inhibitor, batoclimab, for thyroid eye disease. J Clin Endocrinol Metab. (2023) 108:3122–34. doi: 10.1210/clinem/dgad381 37390454 PMC10655547

[140] Suzhou Suncadia Biopharmaceuticals Co. Ltd . A single-arm, open-label, prospective phase II study to evaluate the efficacy and safety of SHR-1314 by subcutaneous injection in active moderate to severe Graves’ orbitopathy patients (2024). Available online at: https://clinicaltrials.gov/study/NCT05394857 (Accessed June 15, 2026).

[141] Builes-Montaño CE Román-González A Arenas-Quintero HM Aristizábal-Henao N Cabarcas-Solano MDS Camargo González J . Pharmacologic therapies for active moderate-to-severe TED: A comprehensive systematic review. Clin Med Insights Endocrinol Diabetes. (2026) 19:11795514261426446. doi: 10.1177/11795514261426446 41783234 PMC12953964

[142] Shah R Adamson SE Jasim S . Management aspects of medical therapy in Graves disease. Endocr Pract. (2025) 31:536–46. doi: 10.1016/j.eprac.2024.12.012 39701285 PMC12005956

[143] Xie P Shen L Peng R Wang Y Yin Q Chen X . Effects of low-dose methotrexate with methimazole in patients with Graves' disease: Results of a randomized clinical trial. J Clin Endocrinol Metab. (2025) 110:489–97. doi: 10.1210/clinem/dgae472 38994582

[144] Lanzolla G Sabini E Leo M Menconi F Rocchi R Sframeli A . Statins for Graves' orbitopathy (STAGO): a phase 2, open-label, adaptive, single centre, randomised clinical trial. Lancet Diabetes Endocrinol. (2021) 9:733–42. doi: 10.1016/S2213-8587(21)00238-2 34592164

[145] Kauppinen-Mäkelin R Karma A Leinonen E Löyttyniemi E Salonen O Sane T . High dose intravenous methylprednisolone pulse therapy versus oral prednisone for thyroid-associated ophthalmopathy. Acta Ophthalmol Scand. (2002) 80:316–21. doi: 10.1034/j.1600-0420.2002.800316.x 12059873

[146] Douglas RS Dailey R Subramanian PS Barbesino G Ugradar S Batten R . Proptosis and diplopia response with teprotumumab and placebo vs the recommended treatment regimen with intravenous methylprednisolone in moderate to severe thyroid eye disease: A meta-analysis and matching-adjusted indirect comparison. JAMA Ophthalmol. (2022) 140:328–35. doi: 10.1001/jamaophthalmol.2021.6284 35175308 PMC8855315

[147] Dalmas F Foing C Darrason G Varoquaux A Mazodier K Muller R . Long-term tocilizumab therapy in patients with corticosteroid-resistant active moderate-to-severe thyroid eye disease. Eur J Endocrinol. (2025) 193:613–21. doi: 10.1093/ejendo/lvaf227 41208406

[148] Mohammadi SO Ray C . Advancing the therapeutic frontier in thyroid eye disease. touchREV Endocrinol. (2025) 21:12–4. doi: 10.17925/EE.2025.21.2.2 41246121 PMC12616671

[149] Lee V Salvi M Eckstein A Dayan C Meeran K . Horizon scanning from panel discussions at the EUGOGO Global TED Forum 2025 London. Eye (Lond). (2026) 40:441–2. doi: 10.1038/s41433-025-04197-z 41501285 PMC12957360

[150] Madgar S Simon GB Priel A Sagiv O Landau-Prat D Cukierman-Yaffe T . Thyroid-stimulating immunoglobulins as a reliable predictive blood test for thyroid eye disease. Indian J Ophthalmol. (2026) 74:129–32. doi: 10.4103/IJO.IJO_1035_24 41460142 PMC12867296

[151] Fang L Ning Q Wu C Liu D Ning J . N-phenethylacetamide, diaminopimelic acid, and Gly-Val as high-performance serum biomarkers for diagnosing untreated Graves' disease: an LC-MS-based metabolomics study. Front Endocrinol (Lausanne). (2025) 16:1707049. doi: 10.3389/fendo.2025.1707049 41306435 PMC12643872

[152] Qian W Zhu T Liu J Wei Y Yang L Fang L . Integrative transcriptomic profiling and machine learning reveal hypoxia-associated molecular signatures for precision diagnosis in thyroid eye disease. Hum Genomics. (2025) 19:140. doi: 10.1186/s40246-025-00860-4 41287009 PMC12642099

[153] Leszczyńska D Pomichter B Szelachowska M Buczyńska-Backiel A Adamska A Popławska-Kita A . Noninvasive biomarkers for Graves' orbitopathy: Clinical relevance of circulating PAI-1, TGF-β and IGF-1R. Clin Ther. (2026) 26:S0149-2918(26)00137-2. doi: 10.1016/j.clinthera.2026.04.019 42191487

[154] Chen Y Yang Y Yang S Yuan W Jiang L Wei R . Extracellular vesicles in Graves' disease and Graves' orbitopathy: immunoregulatory mechanisms, biomarkers, and therapeutic potentials. Front Immunol. (2026) 17:1762376. doi: 10.3389/fimmu.2026.1762376 41694359 PMC12901441

[155] Elia G Ferrari SM Ragusa F Balestri E Botrini C Colapietra F . Nanoparticles in thyroid autoimmunity: Diagnostic and therapeutic applications. J Clin Med. (2026) 15:1428. doi: 10.3390/jcm15041428 41753116 PMC12941794

[156] Wang L Wang J Xu H Sun Y Zhou X Chen Y . Vitamin D dynamics predict treatment response to intravenous glucocorticoids in thyroid-associated ophthalmopathy: a retrospective cohort study. Front Immunol. (2026) 17:1778702. doi: 10.3389/fimmu.2026.1778702 42093983 PMC13139022

[157] Yang X Liu J Guo Q Xu Y Zhao H Shao J . Serum free fatty acid profiles as novel biomarkers for disease activity in Graves' orbitopathy. J Endocrinol Invest. (2026). doi: 10.1007/s40618-026-02913-4 42171675

[158] Wang X Zhang Z Wu H Wang Y Su M Sun X . Predictive value of tear lipidomics biomarkers for TAO activity and relationship with clinical characteristics. Exp Eye Res. (2026) 265:110901. doi: 10.1016/j.exer.2026.110901 41643837

[159] Salvi M . What has changed in thyroid eye disease in the last five years (2020-2025). Eur Thyroid J. (2026) 15:ETJ250363. doi: 10.1530/ETJ-25-0363 41627406 PMC12921392

[160] Burch HB Perros P Bednarczuk T Cooper DS Dolman PJ Leung AM . Management of thyroid eye disease: a consensus statement by the American Thyroid Association and the European Thyroid Association. Eur Thyroid J. (2022) 11:e220189. doi: 10.1530/ETJ-22-0189 36479875 PMC9727317

[161] Lei C Jin J Li R Yip CC Chong KKL Smith TJ . Management of thyroid eye disease: a comparison between three recent clinical guidelines. Ophthalmol Ther. (2026) 15:985–99. doi: 10.1007/s40123-026-01326-z 41697453 PMC12976230

